# BioWF: A Naturally‐Fused, Di‐Domain Biocatalyst from Biotin Biosynthesis Displays an Unexpectedly Broad Substrate Scope

**DOI:** 10.1002/cbic.202200171

**Published:** 2022-07-13

**Authors:** Shona M. Richardson, Peter J. Harrison, Michael A. Herrera, Menglu Wang, Rebecca Verez, Gustavo Perez Ortiz, Dominic J. Campopiano

**Affiliations:** ^1^ School of Chemistry University of Edinburgh David Brewster Road Edinburgh EH9 3FJ UK; ^2^ Diamond Light Source Ltd. Harwell Science & innovation Campus Didcot OX11 0DE UK

**Keywords:** aminoketones, adenylate, biocatalysis, fusion, pyridoxal 5′-phosphate

## Abstract

The carbon backbone of biotin is constructed from the C_7_ di‐acid pimelate, which is converted to an acyl‐CoA thioester by an ATP‐dependent, pimeloyl‐CoA synthetase (PCAS, encoded by BioW). The acyl‐thioester is condensed with ʟ‐alanine in a decarboxylative, Claisen‐like reaction to form an aminoketone (8‐amino‐7‐oxononanoic acid, AON). This step is catalysed by the pyridoxal 5’‐phosphate (PLP)‐dependent enzyme (AON synthase, AONS, encoded by BioF). Distinct versions of *Bacillus subtilis* BioW (*Bs*BioW) and *E. coli* BioF (*Ec*BioF) display strict substrate specificity. In contrast, a BioW‐BioF fusion from *Corynebacterium amycolatum* (*Ca*BioWF) accepts a wider range of mono‐ and di‐fatty acids. Analysis of the active site of the *Bs*BioW : pimeloyl‐adenylate complex suggested a key role for a Phe (F192) residue in the *Ca*BioW domain; a F192Y mutant restored the substrate specificity to pimelate. This surprising substrate flexibility also extends to the *Ca*BioF domain, which accepts ʟ‐alanine, ʟ‐serine and glycine. Structural models of the *Ca*BioWF fusion provide insight into how both domains interact with each other and suggest the presence of an intra‐domain tunnel. The *Ca*BioWF fusion catalyses conversion of various fatty acids and amino acids to a range of AON derivatives. Such unexpected, natural broad substrate scope suggests that the *Ca*BioWF fusion is a versatile biocatalyst that can be used to prepare a number of aminoketone analogues.

## Graphical abstract

**A BioWF fusion** biocatalyst can convert various fatty acid substrates to α‐oxoamine (AON) products. The ATP‐dependent BioW domain catalyses formation of an acyl‐CoA thioester intermediate. The pyridoxal 5’‐phosphate (PLP)‐dependent BioF domain then accepts various amino acids and catalyses the formation of a range of aminoketone analogues.

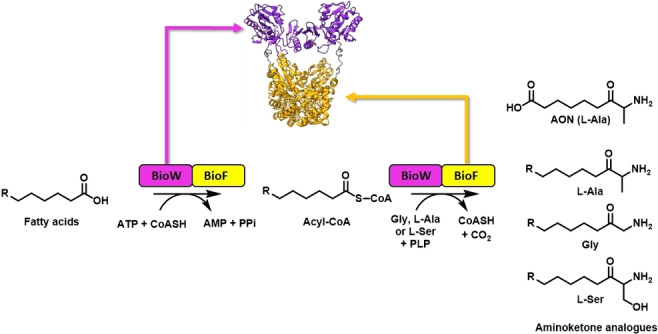

## Introduction

Enzyme fusions are bi‐/multi‐functional biocatalysts that can complete a series of sequential/cascade reactions to form a desired product. Such multi‐step reactions are typically found in biosynthetic pathways and can generate natural products of stunning complexity. An increase in enzymatic cascade efficiency results through close proximity of active sites, coupled with a high local concentration of substrates. This also prevents the build‐up of intermediates that could inhibit enzymatic activity.^[1]^ Examples of natural fusions include the fatty acid synthases (FAS), polyketide synthases (PKS) and non‐ribosomal peptide synthetases (NRPS). These multidomain megasynthases adopt modular assembly lines composed of varying enzymatic domains that can produce a myriad of primary metabolites and secondary natural products.[^2^, ^3^, ^4^, ^5^] Despite their abundance in nature, there is a growing use of synthetically designed fusion systems, achieved by expressing multiple genes as a single transcript.[^6^, ^7^, ^8^] One major drawback of these “beads‐on‐a‐string” systems is linker design, with few investigations into their structure and function to date.^[9]^ By exploiting pre‐existing natural fusions this need for linker understanding is not required, with the fusion systems having undergone extensive evolutionarily optimisation. Therefore, natural multi‐domain fusion systems are ideal candidates for biocatalytic transformations.

Biotin is an unusual bicyclic vitamin constructed through a series of interesting reactions. Biotin production can be divided into two stages. Firstly, formation of an unusual pimeloyl‐thioester from the C_7_ pimelic di‐acid, with the acyl chain linked to either CoASH or an acyl carrier protein (ACP). Two pathways are known for the biosynthesis of this acyl‐thioester with each using a pair of coupled enzymes. The more common methyltransferase/hydrolase (BioC/BioH) pathway is found in *E. coli* and hijacks fatty acid biosynthesis to generate pimeloyl‐ACP. In contrast, the much rarer BioI and BioW‐dependent routes were discovered in *Bacillus subtilis*.[^10^, ^11^, ^12^] The BioI and BioW can act together to produce pimeloyl‐thioesters from long chain fatty acid precursors. Surprisingly, the *B. subtilis* BioI is a cytochrome P450 that oxidatively cleaves long chain acyl‐ACPs, forming the pimeloyl‐ACP intermediate.[^13^, ^14^] Alternatively, *B. subtilis* also encodes a pimeloyl‐CoA synthetase (PCAS, BioW) that catalyses direct formation of pimeloyl‐CoA in an ATP‐dependent reaction.[^15^, ^16^] The genes encoding these proteins reside in the bio‐operon or biosynthetic gene clusters (BGCs); bioABFCD for *E. coli* and bioWAFDBI for *B. subtilis*.^[17]^

Following pimeloyl‐CoA/ACP production, a conserved four‐enzyme pathway ensues. Pimeloyl‐CoA/ACP is condensed with ʟ‐Ala to form 8‐amino‐7‐oxononanioc acid (AON). This is catalysed by the pyridoxal 5’‐phosphate (PLP)‐dependent α‐oxoamine synthase (AONS, encoded by BioF), through a decarboxylative, Claisen‐like condensation. The AON is then transaminated by the PLP‐dependent 7,8‐diaminononanoic acid (DAN) synthase (BioA) to form diaminononanoic acid (DAN). The ureido ring of dethiobiotin is formed by insertion of CO_2_ between the diamine of DAN, catalysed by the ATP‐dependent dethiobiotin (DTB) synthetase (BioD). The final step of biotin formation incorporates sulfur. This step is catalysed by biotin synthase (BioB), a radical SAM enzyme with two Fe−S clusters. The sulfur originates from a deeply buried [2Fe‐2S]^2+^ cluster, and generates the final thiophene ring.

**Figure 1:**
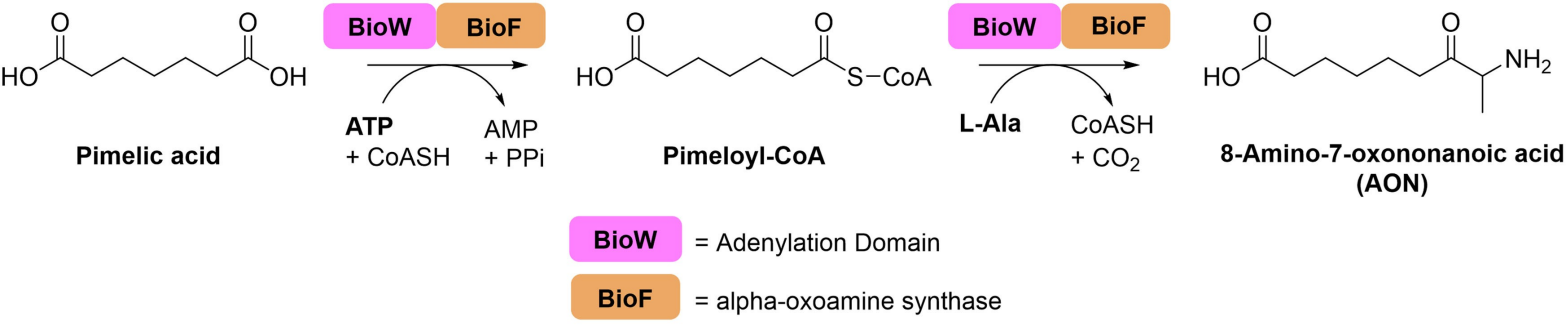
*Ca*BioWF enzymatic reaction taking pimelic acid through the pimeloyl‐CoA intermediate to form the final AON product.

To date, only a few natural fusions of biotin biosynthesis enzymes have been identified. For example, investigations into biotin production in *Arabidoposis thaliana* led to the discovery of a natural fusion of the BioA and BioD genes.^[18]^ The *At*BioAD fusion was shown to catalyse consecutive transamination and ring‐forming reactions. Similarly, the Cronan group reported a natural fusion of the BioW and BioF genes in *Desulfosporosinus orientis* (Figure 1).^[19]^ This study showed that the expressed *D. orientis* BioWF fusion was able to complement a biotin auxotrophic *E. coli* BioF mutant when grown on exogenous pimelic acid. Although providing support that both domains of the fusion were active, the recombinant fusion biocatalyst was not isolated.

To explore the use of fused domains as viable biocatalysts, we identified a BioWF fusion in *Corynebacterium amycolatum* SK46. Here we describe the isolation and characterization of the *Ca*BioWF fusion and show that the recombinant biocatalyst can catalyse the sequential conversion of pimelic acid to pimeloyl‐CoA, followed by formation of the predicted AON product. Furthermore, we discovered that the *Ca*BioWF fusion displays a surprisingly broad substrate scope. The versatile biocatalyst accepts a range of mono‐ and di‐carboxylic acids, activates them as acyl‐CoA thioesters, and then catalyses formation of a range of α‐oxoamines with three different amino acids. Structural and sequence analysis of the *Ca*BioWF fusion suggested that a phenylalanine residue in the predicted BioW active site is the key to its broad activity. The BioF domain of the fusion displays features common to other members of the α‐oxoamine synthase (AOS) family which can accept a range of amino acids. A structural model of the *Ca*BioWF fusion provides insight into how the two domains interact. Our work suggests that other natural fusion enzymes should be explored as potentially useful biocatalysts.

## Results and Discussion

### Cloning an unusual BioWF Fusion from *C. amycolatum* (*Ca*BioWF)

An investigation into bacterial BioW homologues led to the discovery of a predicted natural fusion of the BioW and BioF domains in the organism *C. amycolatum* SK46 (Uniprot: E2MUP3, Figures S1 and S2), a non‐sporulating Gram positive bacterium.^[20]^ The annotated *Ca*BioWF gene was purchased as a synthetic clone from Genscript, cloned in a pET‐28a expression plasmid. Recombinant protein expression and isolation was optimized in *E. coli* BL21 (DE3); however incubation of the purified, recombinant *Ca*BioWF with the reaction components (pimelate, ATP, CoASH, ʟ‐alanine) yielded no product formation (data not shown). However, the addition of purified, active *Bs*BioW to this reaction mixture led to AON production, detected by LC ESI‐MS analysis.^[15]^ This suggested that the *Ca*BioW domain of the fusion was inactive, whereas the *Ca*BioF domain was able to catalyse conversion of the pimeloyl‐CoA (formed by the additional *Bs*BioW) to AON. The N‐terminus was re‐examined using information gained through detailed analysis of *Bs*BioW (Uniprot: P53559, PDB: 5FLL) and the *Aquifex aeolicus* homologue (*Aa*BioW, Uniprot: O67575, PDB: 5TV6) sequences and structures (Figures S3 and S4). This revealed a number of conserved residues preceding the annotated starting Met were missing from the initial *Ca*BioWF (equivalent to Met12 in *Bs*BioW).[^15^, ^16^] The predicted N‐terminus also starts in the middle of key secondary structure (β‐sheet) of *Bs*BioW and therefore is likely to interfere with protein folding. Therefore, the N‐terminal domain was extended and 7 amino acids were inserted through Gibson assembly cloning (Figure S5).

### Characterisation of a fully active *Ca*BioWF fusion

The expression of this new *Ca*BioWF construct was first tested under a series of standard conditions, with protein production observed for each one tested (Figure S6). The protein was subsequently extracted and purified via nickel immobilised affinity chromatography (IMAC), followed by HisTag removal by TEV‐protease and size exclusion chromatography (SEC), characterising the protein as a homodimer. A mass of 67432.45±0.31 was obtained via LC ESI‐MS analysis, corresponding to the untagged predicted mass of 67431.93 Da (Figure S7). After isolation of this new construct with a re‐designed N‐terminus, the activity of the *Ca*BioW domain was once again tested.

### Activity of the BioW domain of the *Ca*BioWF fusion

**Figure 2:**
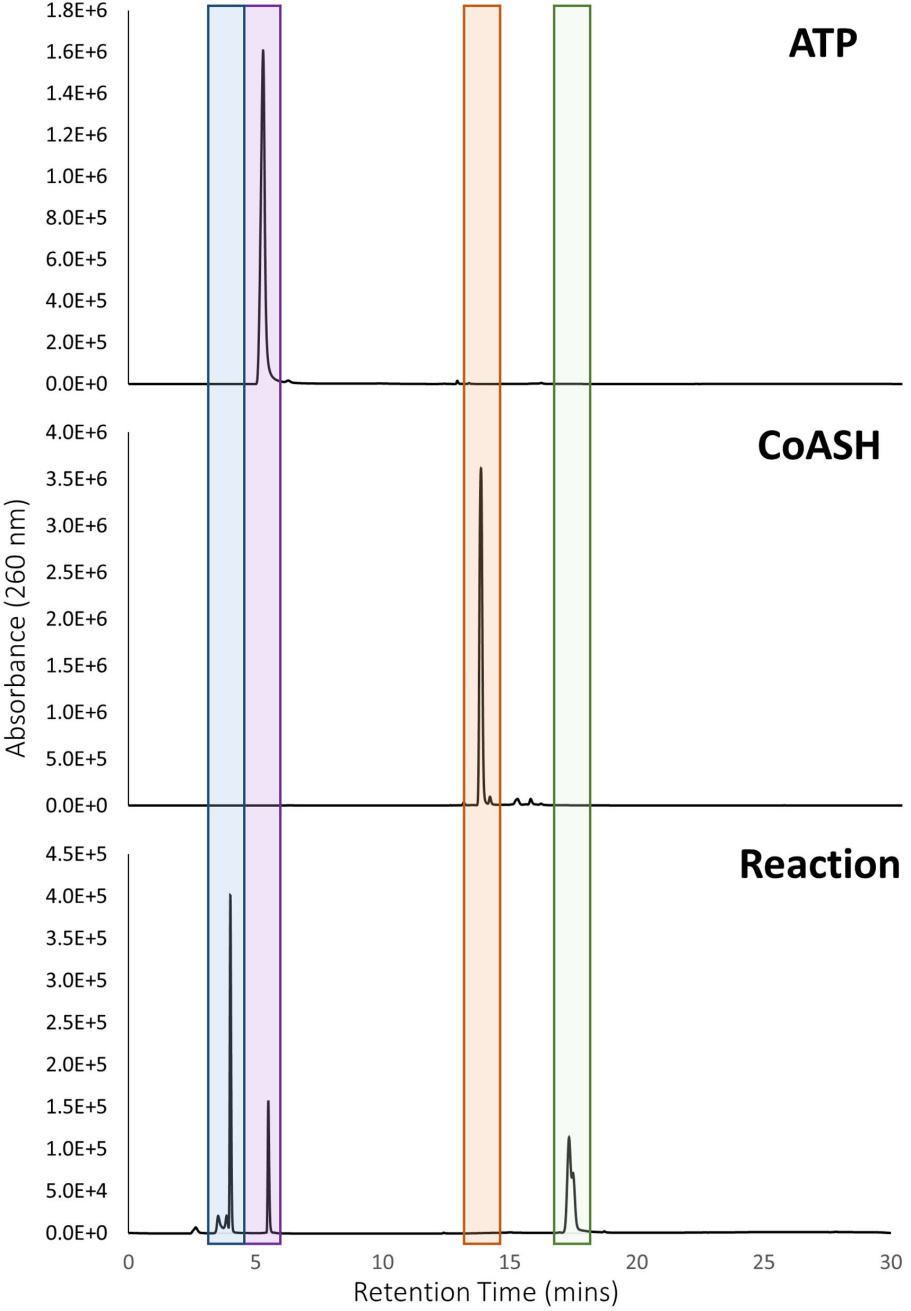
HPLC assay results for *Ca*BioWF enzymatic reaction with DC_7_ pimelic acid, ATP and CoASH leading to the formation of pimeloyl‐CoA (green, 17.3 min) and released AMP (blue, 4.0 min). This is shown with the corresponding ATP (purple, 5.2 min) and CoASH (orange, 13.7 min) standards.

This N‐terminal BioW adenylation domain should catalyse the formation of pimeloyl‐CoA in an ATP‐dependent reaction. Product formation can be monitored using HPLC which can detect the acyl‐CoA thioester at 260 nm. This assay was previously reported by Wang *et al*.^[15]^ The appearance of a peak at 17.3 min in the chromatogram signified the formation of pimeloyl‐CoA, which was later confirmed by FT‐ICR MS, with detection of an ion with *m*/*z*=932.16912 Da, matching the predicted mass of 932.1674 ([M+Na]^+^, C_28_H_46_N_7_O_19_P_3_S (Figure 2 and Figure S8). This confirmed that the re‐engineered N‐terminus recovered the *Ca*BioW activity in the *Ca*BioWF fusion.

The kinetics of the newly “re‐activated” domain was studied using the coupled MesG assay originally reported by Aldrich *et al*. and Webb *et al*.[^21^, ^22^] When *Ca*BioWF (0.1 μM) was incubated with varying amounts of pimelic acid (0 to 1000 μM), ATP and CoASH, an increase in the absorbance at 360 nm was monitored. The kinetic data was fitted with a Michaelis‐Menten curve to give a K_M_ value of 58.6±3 μM, and a k_cat_ of 0.21 s^−1^ for pimelic acid (Figure S9). This analysis confirms that the activity of the *Ca*BioW domain of the fused system is comparable to the *Bs*BioW homologue.

### Activity of the BioF domain of the *Ca*BioWF fusion

**Figure 3:**
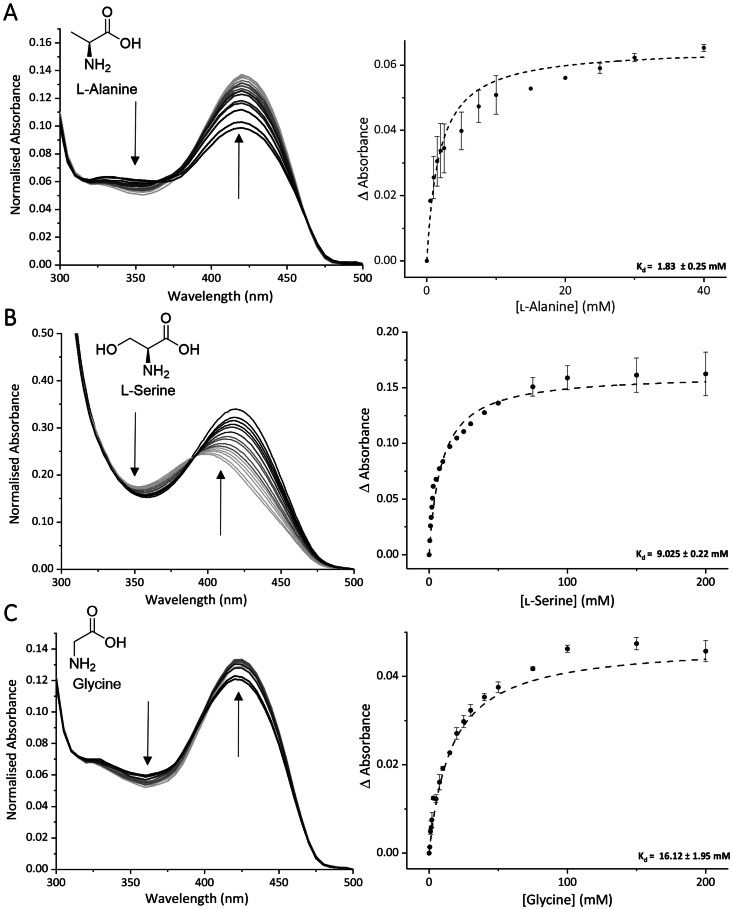
UV‐Vis spectroscopy scan of the *Ca*BioF domain PLP binding monitoring the changes after the addition of A) ʟ‐Ala (0–40 mM) and using the changes in the absorbance at 425 nm, a K_d_ curve was plotted and the dissociation constant of 1.83±0.25 mM calculated. Also, after the addition of B) ʟ‐Ser (0–200 mM) a K_d_ of 9.03±0.22 mM was calculated and for C) Gly (0–200 nm) a K_d_ of 16.12±1.95 mM.

Having confirmed activity of the N‐terminal BioW domain, we next tested the C‐terminal *Ca*BioF domain for its predicted AONS activity. This domain should catalyse the formation of AON from ʟ‐Ala and pimeloyl‐CoA in a PLP‐dependent reaction.^[23]^ Sequence alignments to the previously studied *Ec*BioF identified the conserved, key PLP‐binding residue as Lys484 (equivalent to *Ec*BioF residue Lys236, Figure S10). PLP‐binding can be monitored by changes in the characteristic UV‐Vis spectrum.[^19^, ^23^] Upon titration of ʟ‐Ala (0‐40 mM) into the PLP‐bound *Ca*BioWF enzyme we noted a change in absorbance from 250 nm to 500 nm, indicating the formation of the PLP:ʟ‐Ala external aldimine (Figure 3A). In order to monitor the changes as the reaction proceeds, pimelic acid and CoASH were then titrated into the mixture. With the addition of MgCl_2_ and ATP, the *Ca*BioW domain should produce the pimeloyl‐CoA product, which can then be immediately utilised by the *Ca*BioF domain. This is apparent in the UV‐Vis spectrum, with changes in absorbance being observed up to 5 h after the addition (Figure S11). The binding of the amino acid can be quantified through the calculation of a dissociation constant (K_d_). Again, ʟ‐Ala was titrated into the *Ca*BioWF PLP‐bound mixture at varying concentrations (0–40 mM). The changes in absorbance were measured after each addition and the absorbance at 425 nm used to calculate the K_d_. This gave rise to a K_d_ of 1.83±0.25 mM (Figure 3A).

In addition to the natural substrate ʟ‐Ala, binding of two other amino acids (ʟ‐Ser and Gly) was also observed and binding was quantified with K_d_ values of 9.03±0.22 mM for ʟ‐Ser (Figure 3B) and 16.12±1.95 mM for Gly (Figure 3C). Although the affinity for both of these amino acids is weaker than ʟ‐Ala, both amino acids are still accepted.

### The full *Ca*BioWF reaction

Having observed that the *Ca*BioF domain could bind different amino acids, we next determined if the *Ca*BioWF fusion is able to generate a number of AON analogues. Therefore, a series of full *Ca*BioWF reactions, incubating pimelic acid with ʟ‐Ala, ʟ‐Ser or Gly, were conducted. To study the complete reaction, *Ca*BioWF was incubated with the *Ca*BioW reaction components and ʟ‐Ala at 30 °C for 5 h. The mixture was quenched by the addition of 1 : 1 (v:v) acetonitrile (MeCN) (0.01 % TFA) and the final AON product was analysed by LC ESI‐MS analysis. An ion with *m/z*=188.1282 was observed in the LC ESI‐MS and corresponds to the predicted mass of AON at 188.1280 ([M+H]^+^, C_9_H_18_NO_3_, Figure S12), thereby confirming the cooperation of the two domains in the formation of the AON product.

**Figure 4:**
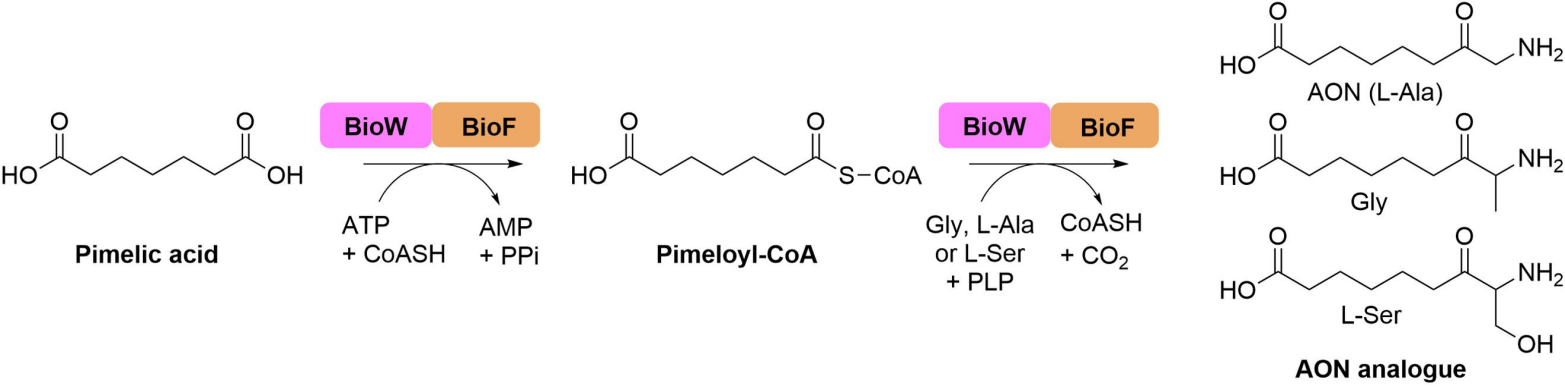
LC ESI‐MS analysis of the full CaBioWF reaction upon incubation with pimelic acid and CoASH leading to first the production of the pimeloyl‐CoA intermediate and then the C−C bond forming reaction either A) Gly, B) ʟ‐Ala leading to the production of AON or C) ʟ‐Ser, each leading to the formation of the corresponding aminoketone product. Product formation was confirmed by LC ESI‐MS analysis.

Using a commercially‐available AON standard, a calibration curve was constructed using HPLC and LC–MS analysis (Figure S13A and B). This allowed the amount of AON to be determined initially in a small‐scale *Ca*BioWF reaction. Two reactions, using either 1 mM or 0.5 mM CoASH, were performed, leading to the production of AON at concentrations of 0.76 mM and 0.79 mM respectively. These values correspond to a conversion 50.6 and 52.6 % (based on pimelic acid). The negligible difference between the two reactions emphasizes the ability of *Ca*BioWF to recycle CoASH. We also explored the utility of *Ca*BioWF at a larger scale. A 10 mL reaction, using 0.5 mM CoASH and 5 μM biocatalyst, resulted in an AON conversion of 51.3 % (0.77 mM) over 5 h, similar to those obtained on a smaller scale (Figure S13C). *Ca*BioWF is therefore a promising biocatalyst with an inbuilt CoASH recycling system.,

We then repeated this analysis using pimelic acid and both ʟ‐Ser and Gly. In both cases the corresponding aminoketone product can be identified, with *m/z*=204.1267 and *m/z*=174.1132 matching the predicted mass of 204.1231 ([M+H]^+^, C_9_H_18_NO_4_) and 174.1125 ([M+H]^+^, C_8_H_16_NO_3_) respectively for the ʟ‐Ser and Gly derived AON derivatives (Figure 4, S12A and C).

### The full *Ca*BioWF substrate scope

Since BioW is the key enzyme that controls the acyl‐chain length of biotin, we expected the equivalent domain of the *Ca*BioWF fusion would only accept pimelic acid as a substrate. This was previously observed for both *Bs*BioW and *Aa*BioW.[^15^, ^16^, ^24^] Having found that the enzyme is active with the native substrate, the *Ca*BioWF was incubated with various di‐acids (DC_6_‐DC_9_) and mono acids (C_6_‐C_10_), using the same reaction conditions and analysis as before. A series of small peaks between 15 and 25 minutes was observed by HPLC, and we expected them to correspond to the acyl‐CoA products ‐ DC_6_ (16.5 min), DC_8_ (18.3 min), DC_9_ (19.1 min), C_6_ (20.1 min), C_7_ (21.2 min), C_8_ (22.1 min), C_9_ (23.4 min) and C_10_ (24.0 min) (Figures S14 and S16). MS analysis of each reaction confirmed the formation of the expected product, with the correct [M+H]^+^ ion observed for each reaction (Figures S15 and S17, except for C_6_‐CoA for which no product ion was observed despite the presence of a visible peak in the HPLC).

The broad substrate scope of *Ca*BioWF was unexpected. Estrada *et al*. noted acyl‐adenylate formation was observed when *Aa*BioW was incubated with DC_6_ and DC_8_ acids, however no acyl‐CoA was formed upon incubation with CoASH, but rather hydrolysis of the acyl‐adenylate was observed leading to acid reformation and AMP release.^[24]^ Therefore, a multiple sequence alignment (*Ca*BioWF, *Bs*BioW and *Aa*BioW) enabled the identification of the corresponding active site residues in *Ca*BioWF (Figure S3).

**Figure 5:**
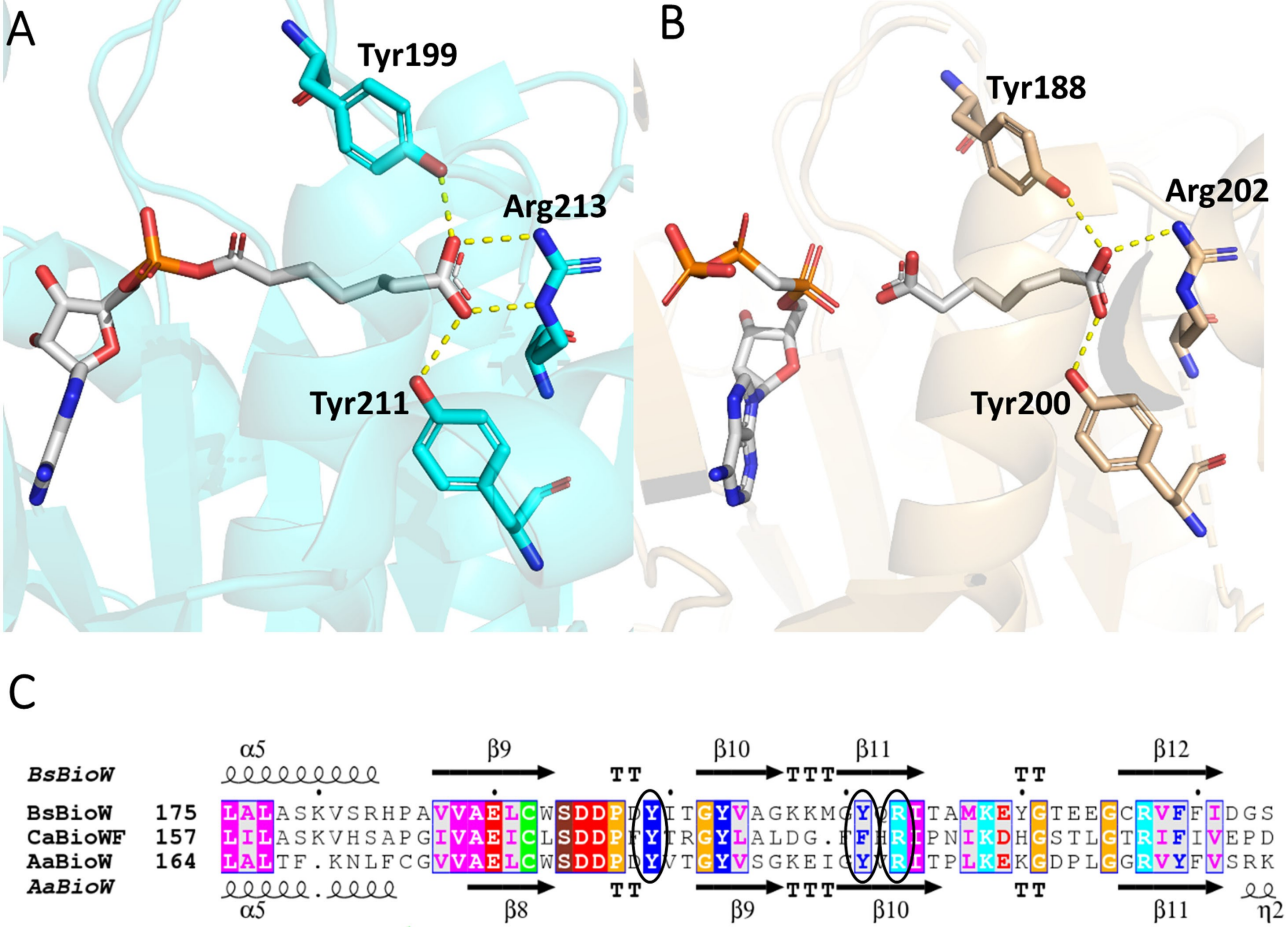
Sequence and structural analysis of the BioW domains A) *Bs*BioW structure (PDB:5FLL) with pimeloyl‐CoA bound, showing interactions with key active site residues Tyr199, Tyr211 and Arg213 and B) *Aa*BioW structure (PDB: 5TV8) with CoASH and pimelate bound showing interactions with active site residues Tyr188, Tyr200 and Arg202. C) Sequence alignment of *Bs*BioW, *Ca*BioWF and *Aa*BioW, with the corresponding predicted active site residues circled (Phe192 corresponds to *Bs*BioW Tyr211 and *Aa*BioW Tyr200).

Three active site residues (Tyr199, Tyr211 and Arg213 in *Bs*BioW) were shown to form key H‐bond and electrostatic interactions with the terminal carboxyl group of the bound pimeloyl‐CoA. By aligning *Ca*BioWF with *Bs*BioW and *Aa*BioW, one of the key Tyr residues is a Phe (Phe192), which corresponds to the Tyr199 in *Bs*BioW and Tyr188 in *Aa*BioW (Figure 5). Rational engineering generated a *Bs*BioW Y199F mutant that was able to accept a range of mono‐acids and di‐acids.^[15]^ Therefore we hypothesised that the equivalent Phe192 residue in the *Ca*BioWF fusion is the reason for the observed broad substrate range.

To test this hypothesis, a *Ca*BioWF F192Y fusion mutant was prepared. The purified fusion mutant biocatalyst was incubated under the same conditions as the wild type *Ca*BioWF version. LC ESI‐MS analysis of the enzyme gave a mass of 68567±3.19 Da, which corresponds to the predicted mass of 68682.23 Da, equivalent to a mass difference of +16 Da (F ‐ Y mutation) and −131 Da (loss of N‐terminal Met) compared to the wild type *Ca*BioWF (Figure S18). The *Ca*BioWF F192Y mutant was then incubated with the series of acid substrates (DC_6_‐DC_9_ and C_6_‐C_9_). In contrast to the wild type enzyme, acyl‐CoA product formation was only observed for the natural DC_7_ pimelic acid substrate, with no other products detected via HPLC analysis (Figure S19). This result provides evidence to support the key role that the Phe192 residue plays in allowing the *Ca*BioWF fusion to accept a range of acid substrates.

### The full *Ca*BioWF coupled biocatalytic reaction

Having established the broad substrate scope of the *Ca*BioWF fusion, we examined the synthetic potential of this biocatalyst by screening combinations of various fatty acid and amino acid substrates . The *Ca*BioWF was incubated with PLP, ʟ‐Ala and acids (DC_6_−DC_9_ and C_6_−C_9_). We also included one example with ʟ‐Ser and Gly. Gratifyingly, all of the predicted AON products were observed by LC ESI‐MS (Figure S20, Table 1).

**Table 1:** Tabulation of the predicted and observed *m/z* values for the AON analogues, formed upon incubation of *Ca*BioWF with various combinations of fatty acids and amino acids.

Compound^[a]^	*m/z* Predicted	*m/z* Observed^[b]^
DC_6_+ʟ‐Ala	174.1125	174.0664
DC_7_+ʟ‐Ala	188.1281	188.1282
DC_8_+ʟ‐Ala	202.1438	202.1433
DC_9_+ʟ‐Ala	216.1594	216.1598
C_6_+ʟ‐Ala	144.1387	144.1383
C_7_+ʟ‐Ala	158.1539	158.1527
C_8_+ʟ‐Ala	172.1696	172.1693
C_9_+ʟ‐Ala	186.1852	186.1856
DC_7_+ʟ‐Ser	204.1230	204.1267
DC_7_+Gly	174.1125	174.0664
C_7_‐Br+ʟ‐Ala	236.0686	236.0664
	238.0664	238.06625
C_6_‐Me+ʟ‐Ala	172.1696	172.1675

Encouraged by this result, a pair of unusual carboxylic acids (7‐bromoheptanoic acid and 6‐methylhexanoic acid) were incubated with the enzyme. In both cases the corresponding aminoketone product was formed with ʟ‐Ala and was observed in the mass spectrum. In particular the formation of the AON derived from 7‐bromoheptanoic highlights the synthetic utility of this novel enzyme, as the C−Br can be used as a handle to introduce further diversity (Figure S21, Table 1).

We conclude that the unexpected broad substrate scope extends between both domains of the natural *Ca*BioWF fusion and thus this biocatalyst can be used to prepare a number of AON analogues.

### Structural analysis of the *Ca*BioWF fusion

*Ca*BioWF was accurately modelled as a homomeric complex using the deep learning architecture ColabFold;[^25^, ^26^] this complex was subsequently relaxed and studied in a 10 ns molecular dynamics simulation (MDS, see Supporting Information for detailed analysis (Figures S22‐29)). In summary, both *Ca*BioW and *Ca*BioF domains are expected to contribute towards homodimerisation; the predicted complex could maintain its structural integrity when scrutinised by MDS. *Ca*BioWF also demonstrates moderate conformational flexibility, owing partly to the disordered linker(s) connecting the *Ca*BioW and *Ca*BioF domains. Intriguingly, the relative orientation and proximity of the *Ca*BioW and *Ca*BioF active sites implies the existence of a ∼5 nm molecular “tunnel”, permitting the facile diffusion of pimeloyl‐CoA from *Ca*BioW to *Ca*BioF. Taken together, this *in silico* study afforded valuable insight into the didomain, homomeric architecture of *Ca*BioWF, making it an attractive target for further structural studies.

## Conclusion

Here we demonstrate that fusion enzymes can be useful biocatalytic tools with the ability to perform multiple, consecutive transformations in one‐pot. This work has explored a novel bifunctional fusion enzyme (*Ca*BioWF) that displays an inherently broad substrate scope for the formation of a range of synthetically useful aminoketone building blocks.

The recombinant *Ca*BioWF has been shown to catalyse the consecutive steps of pimeloyl‐CoA formation, followed by condensation with ʟ‐Ala to form AON. The individual *Ca*BioW and *Ca*BioF domains within the fusion biocatalyst were assayed. Analysis of the *Ca*BioW domain revealed a natural substrate promiscuity that is unprecedented when compared with other BioW homologues. The domain was able to convert a series of mono‐ and di‐acid (C_6_−C_10_ and DC_6_−DC_9_) substrates, forming the corresponding acyl‐CoA products. A divergent Phe residue in the predicted *Ca*BioW active site was proposed to be responsible for this broad substrate scope. Conversion of this Phe to Tyr restored the narrow substrate specificity exhibited by single domain BioW homologues.

In contrast to the single domain BioF homologues, the PLP‐dependent *Ca*BioF domain of the fusion also displayed an expanded substrate scope. It was able to bind ʟ‐Ala, ʟ‐Ser and Gly and catalyse formation of the corresponding AON derivatives. By combining both activities, the *Ca*BioWF fusion biocatalyst was able to condense a range of fatty acid substrates (C_6_‐C_9_ and DC_6_‐DC_9_) with ʟ‐Ala to generate eight aminoketone derivatives. Furthermore, the head‐group could be diversified by condensation with ʟ‐Ser and Gly. A further two aminoketones were generated from unusual fatty acid substrates. In total, twelve aminoketone products were produced, but the inherent properties of this fusion suggest that it has even greater synthetic potential. Structural insights into this unusual *Ca*BioWF fusion biocatalyst were obtained by predictive modelling and simulation. This revealed a putative tunnel that may enable facile diffusion of the pimeloyl‐CoA product from the BioW to the BioF domain. The characterisation of this unique di‐domain biocatalyst should encourage the discovery of other natural fusions embedded in the BGCs of other genomes. This will be facilitated by the continued advances in sequencing and metagenomics.

## Experimental Section

**Cloning and expression of** ***Ca*** **BioWF in** ***E. coli***: The originally purchased synthetic *Ca*BioWF gene was re‐cloned into pET28α, adding 7 amino acids (MSTYSIR, Figure S4) to the N‐terminus using the following primers. BioWF For (NcoI) – GAGATATACC ATGGGCAGTA CCTACAGCA, BioWF Rev (BamHI) – TTTCGGATCC CAGCAGGCCC pET28α For (BamHI) ‐ CTGCTGGGAT CCGAAAACCT GTATTTTC, pET28α Rev (NcoI) – GTAGGTACTG CCCATGGTAT ATCTCCTTCTT. After PCR, both PCR products were incubated together with the Gibson master mix at 50 °C before being transformed into C2987 cells. This generated the plasmid pET‐28a/*Ca*BioWF which was used to transform BL21 (DE3) cells. Expression from this construct was optimised (Figure S6) and suitable conditions were chosen for a larger scale (typically 2 litres of LB broth supplemented with kanamycin). The recombinant *Ca*BioW was expressed using 0.5 mM isopropyl β‐D‐thiogalactoside (IPTG) at 20 °C for 18 h. Cells were harvested by centrifugation and stored at −20 °C.

**Purification of** ***Ca*** **BioWF variants from** ***E. coli***: Cell pellets were defrosted on ice and then resuspended in the corresponding lysis buffer (sodium phosphate pH 7.5 (20 mM), NaCl (500 mM), imidazole (10 mM), PLP (25 μM)). Around 10 mL of resuspended pellets was then sonicated for 10 min in 30 s intervals to lyse the cells. Cell free extract was obtained by centrifugation at 10,000 rpm for 45 min and the supernatant was filtered (0.45 μM filter). The protein was purified from this cell lysate. Each purification step was completed at 4 °C or on ice. The first purification step consisted of nickel IMAC. The protein was loaded onto a 5 mL HisTrap HP column (GE Healthcare) attached to an ÄKTA Purifier system and protein elution monitored at 280 nm. The filtered cell lysate was loaded onto the column at 3 mL/min and washed with the corresponding lysis buffer for 3 column volumes (CVs) to remove any unbound protein. The protein eluted with an increasing imidazole gradient (10 mM–300 mM) over 20 CVs using an increasing gradient of the elution buffer (Sodium phosphate pH 7.5 (20 mM), NaCl (100 mM), imidazole (300 mM), PLP (25 μM)). The eluted protein was concentrated down to ∼1–5 mL using a molecular weight cut‐off concentrator (MWCO, satorius). The HisTagged protein was incubated with pure TEV protease (1 : 10 ratio based off protein concentration (mg/mL)) and dialysed against the size exclusion chromatography (SEC) buffer 2 L (HEPES pH 8 (20 mM), NaCl (100 mM), PLP (25 μM), 10 % glycerol) for 2 h at 4 °C. Following dialysis, the protein was injected onto a 5 mL HisTrap column (Nickel IMAC) and the untagged enzyme was collected with the flow through over 5 CVs. This removed the TEV and any remaining tagged protein which remained bound to the columns and then eluted off the column using the elution buffer (300 mM imidazole). The collected untagged protein was concentrated down using a MWCO concentrator to a final volume of 1–5 mL. This was then loaded onto a pre‐equilibrated (SEC buffer, 1 CV) HiLoad 16/60 Superdex S200 column (120 mL) from GE healthcare. The protein was eluted over 1 CV at a flow rate of 0.5‐1 mL/min with elution monitored at 280 nm. The fractions containing *Ca*BioWF (Figure S7) were combined, concentrated and flash frozen before being stored at −80 °C.

**MesG Assay of BioW domain**: The activity of BioWF was determined using the 7‐methyl‐6‐thioguanosine (MesG) assay. Each well contained a final concentration of Tris HCl pH 8 (50 μM), NaCl (100 mM), MgCl_2_ (10 mM), triscarboxyethylphosphine (TCEP, 1 mM), CoASH (1 mM), ATP (1 mM), 7‐methyl‐6‐thioguanosine (MesG, Berry & Associates, 0.5 mM), inorganic pyrophosphatase (PPase from baker's yeast, Sigma‐Aldrich, 0.03 U), bacterial purine nucleoside phosphorylase (PNP, Sigma‐Aldrich, 1 U) and *Ca*BioWF (0.1 μM) enzyme. After the addition of the components the plate was pre‐incubated at 30 °C for 15 min. The reaction was then initiated by the addition of pimelic acid (0 to 1000 μM). The increase in absorbance at 360 nm resulting from the enzymatic conversion of MesG to 7‐methyl‐6‐thioguanine was monitored over 1 hr on a BioTek Synergy HT plate reader with Costar 96‐well UV‐transparent plates. The data from the first 10 min was analysed using the Michaelis‐Menten model and a nonlinear regression fit on origin gave values of K_M_ and k_cat_.

**BioWF acyl‐CoA formation reaction**: The *Ca*BioW domain was analysed by acyl‐CoA formation which can be monitored by HPLC. Reactions contained *Ca*BioWF (5 μM), TCEP (0.2 mM), ATP (1 mM), CoASH (1 mM) and mono‐ or di‐acids (DC_6_−DC_9_ and C_6_−C_10_, 1.5 mM) in buffer (Tris ⋅ HCl (25 mM, pH 8), NaCl (50 mM), MgCl_2_ (5 mM)) in a final volume of 1 mL and heated at 30 °C for 5 h.

**Acyl‐CoA HPLC assay**: Reactions were quenched with a 1 : 1 (v/v) ratio of MeCN. 10 μL of the sample was then injected onto Luna 5uM C18 RP‐HPLC column (100 Å, 250×4.60 mm, Phenomenex), and eluted with 95 % water (0.1 % TFA (trifluoroacetic acid), v/v) for 5 min followed by a 25 min gradient from 5 % to 55 % acetonitrile (0.1 % TFA, v/v)/water (0.1 % TFA, v/v) which was maintained for 2 min at 260 nm.

**Acyl‐CoA MS analysis**: Reactions were quenched using a 1 : 1 (v/v) of MeCN with 0.01 % formic acid and centrifuged at 17000×g. 5 μL of the supernatant was injected into a ProSwift C4‐RP‐5H (Thermo) column coupled to a FT‐ICR MS (Daltonics 12T SolariX). The LC gradient (acetonitrile/water) ran from 0 % to 60 % acetonitrile with 0.1 % formic acid over 25 min before being increased to 90 % acetonitrile for 3 min. EICs and masses were determined on DataAnalysis V4.3 software.

***Ca*** **BioWF full reaction**: Reactions contained *Ca*BioWF (5 μM), TCEP (0.2 mM), ATP (1 mM), CoASH (1 mM) and mono‐ or di‐Acids (DC_6_−DC_9_ and C_6_−C_10_, 1.5 mM) and amino acid (ʟ‐Ala, ʟ‐Ser or Gly) in buffer (Tris ⋅ HCl (25 mM, pH 8), NaCl (50 mM), MgCl_2_ (5 mM)) in a final volume of 1 mL and heated at 30 °C for 5 h.

***Ca*** **BioWF full reaction MS analysis**: Reactions were quenched using a 1 : 1 (v/v) of MeCN with 0.01 % TFA and centrifuged at 17000×g. 5 μL of the supernatant was injected into a Phenomenex Kinetex 5 μm C18 100 Å column coupled to a micrOTOF II (Bruker). The LC gradient ran from 5 % acetonitrile and 95 % water with 0.1 % formic acid to 95 % acetonitrile over 10 min. EICs and masses were determined on DataAnalysis V4.3 software.

**PLP binding UV‐Vis spectroscopy**: The UV‐Vis analysis was carried out on a Varian Cary® UV‐Vis spectrophotometer. Excess PLP was removed through desalting using a PD10 column (GE healthcare) and the enzyme exchanged into the storage buffer minus PLP. Assays were carried out in 1 cm pathlength quartz cuvettes and baseline correction was carried out before acquiring spectra.

***Ca*** **BioWF changes in PLP binding**: The protein was diluted to ∼20 μM and an initial PLP bound enzyme curve was obtained by scanning from 250 nm to 500 nm. ʟ‐Ala was titrated from a 1 M stock to a final concentration of 10 mM. The mixture was left to allow time for binding and after 30 min a second scan was taken. Next pimelic acid and CoASH (0.5 mM) were added, alongside MgCl_2_ (10 mM) and ATP (1 mM). The changes in absorbance were analysed over 5 h, showing the changes in absorbance as the *Ca*BioW domain formed pimeloyl‐CoA which was then utilized by the *Ca*BioF domain.

**PLP enzyme‐amino acid dissociation constants (K_d_)**: The protein was diluted to ∼20 μM and the amino acid substrates were titrated from a 1 M stock solution to varying final concentrations (0–100 mM). The spectra were normalized against the 280 nm peak to account for dilution of the sample with addition of substrate solution. Changes in the absorbance maximum of the ketoenamine peak (∼425 nm) were plotted and fitted with a hyperbolic saturation curve using Origin software.

**AON calibration curve**: To estimate the relative abundance of AON produced by *Ca*BioWF, a calibration curve was created using AON hydrochloride (Cayman Chemical) and analysed by LCMS, monitoring the mass of 188.1280 ([M+H]^+^, C_9_H_18_NO_3_). A 10 mg/mL stock solution of AON (44.7 mM) was made in DMSO. This was diluted to 1 mM in buffer (Tris ⋅ HCl (25 mM, pH 8), NaCl (50 mM), MgCl_2_ (5 mM)). Further dilutions made calibration solutions of 0, 2, 5, 10, 20, and 50 μM in triplicate. The LC‐MS used has an Agilent 1200 Series HPLC and a Bruker Daltonics micrOTOF‐II High performance Time‐of‐flight mass spectrometer (TOF‐MS). 5 μL of each calibration solution was injected onto a Phenomenex Kinetex C18 reverse phase column (50×2.1 mm, 2.6 μm particle size) at a flow rate of 0.2 mL/min. The elution was conducted as follows (5% MeCN for 0.5 mins, 100% MeCN for 5 mins, then maintained at 100% for 2 mins, then at 7 mins return to 5% MeCN, followed by re‐equilibration for 2.5 mins). The retention time for AON using this method is 1.67 mins. EICs and masses were determined on DataAnalysis V4.3 software. The area under the curve (AUC) was used to construct a calibration curve of concentration of AON (Figure S13).

***Ca*** **BioWF full reaction for quantitative analysis**: Reactions contained *Ca*BioWF (5 μM), TCEP (0.2 mM), ATP (1 mM), CoASH (1 mM or 0.5 mM), pimelic acid (1.5 mM) and ʟ‐alanine (1.5 mM) in buffer (Tris ⋅ HCl (25 mM, pH 8), NaCl (50 mM), MgCl_2_ (5 mM)) in final volumes of 1000 and 500 μL respectively and heated at 30 °C for 5 h. Reactions were quenched using 1.7 % TFA (60 μL per 1 mL of reaction) and centrifuged at 17000×g for 10 minutes. The supernatant was diluted (1 : 10, 1 : 20, 1 : 50) for LCMS analysis using the same buffer and loaded onto the LCMS, treating the samples in the same way as the calibration standards.

***Ca*** **BioWF preparative scale reaction and quantitative analysis**: Reactions contained *Ca*BioWF (5 μM), TCEP (0.2 mM), ATP (1 mM), CoASH (0.5 mM), Pimelic Acid (1.5 mM) and ʟ‐alanine (1.5 mM) in buffer (Tris ⋅ HCl (25 mM, pH 8), NaCl (50 mM), MgCl2 (5 mM)) in 10 mL final volume, were heated at 30 °C for 5 h with 180 rpm agitation. Reactions were quenched using 1.7 % TFA (60 μL per 1 mL of reaction) and centrifuged at 17000×g for 10 minutes. The supernatant was diluted (1 : 10, 1 : 20, 1 : 50) for LCMS analysis using the same buffer and loaded onto the LCMS, treating the samples in the same way as the calibration standards. The rest of the reaction was stored at −80 °C.

**Structure prediction**: All structural predictions were performed using ColabFold via AlphaFold2_advanced.ipynb. In brief, a deep multiple sequence alignment (MSA) was generated using MMSeqs2 prior to structure prediction using AlphaFold 2 (structural templates were not utilised for prediction). ColabFold was configured to perform homodimeric prediction, and the output of the AlphaFold 2 structure module was recycled up to 3 times for refinement. For each sequence, a total of 5 models were generated and ranked by Predicted Template Model score (pTM); Predicted Local Distance Difference Test (pLDDT) scores were also computed for each model to evaluate fold‐level confidence. The best ranked model was subsequently relaxed to eliminate steric clashes. Visual inspection was performed in UCSF ChimeraX 1.3^[27]^ and PyMOL 2.4.

**Evolutionary conservation analysis**: Evolutionary conservation analysis was performed using the ConSurf[^28^, ^29^] server configured to build MSAs using MAFFT. 150 homologous sequences with identities ranging from 30–95 % were compiled from UNIREF90 using the HMMER search algorithm. Conservation scores were calculated via the Bayesian method and visualised using UCSF ChimeraX 1.3.

**Molecular dynamics simulation**: Simulations were performed using GROMACS 2021.4.^[30]^ Protein charges were computed using CHARMM36 all‐atom forcefield.^[31]^ The model was solvated in TIP3P water in a cubic box, and the net protein charge was counterbalanced using simulated sodium ions. The system was energy‐minimised by sequential steepest descent/conjugate gradient descent and equilibrated to 300 K and 1 bar using V‐Rescale thermostat/Berendsen barostat. Following a 10 ns (5×10^6^ time steps) production MD, the trajectory was recentred with additional rotational and translational fitting. Further analysis was performed in GROMACS using *gmx gyrate*, *gmx hbond*, *gmx rms* and *gmx rmsf*. UCSF Chimera 1.16^[32]^ was used for trajectory visualisation and for computing pairwise RMSDs.

## Conflict of interest

The authors declare no conflict of interest.

1

## Supporting information

As a service to our authors and readers, this journal provides supporting information supplied by the authors. Such materials are peer reviewed and may be re‐organized for online delivery, but are not copy‐edited or typeset. Technical support issues arising from supporting information (other than missing files) should be addressed to the authors.

Supporting Information

## Data Availability

The data that support the findings of this study are available in the supplementary material of this article.
