# Supplementary material for: BioWF: A Naturally‐Fused, Di‐Domain Biocatalyst from Biotin Biosynthesis Displays an Unexpectedly Broad Substrate Scope
Source: Chembiochem. 2022 Jul 13;23(17):e202200171. doi: 10.1002/cbic.202200171 (PMC9544090; doi:10.1002/cbic.202200171)
Supplement: Supplementary file 1 — Supporting Information [file CBIC-23-0-s001.pdf]

ChemBioChem

Supporting Information

BioWF: A Naturally-Fused, Di-Domain Biocatalyst from Biotin Biosynthesis Displays an Unexpectedly Broad Substrate Scope

Shona M. Richardson, Peter J. Harrison, Michael A. Herrera, Menglu Wang, Rebecca Verez, Gustavo Perez Ortiz, and Dominic J. Campopiano*

Supplementary Information

BioWF: A naturally-fused, di-domain biocatalyst from biotin biosynthesis displays an unexpectedly broad substrate scope.

Shona M. Richardson[a], Peter J. Harrison[a,b], Michael A. Herrera[a], Menglu Wang[a], Rebecca Verez[a], Gustavo Perez Ortiz[a] and Dominic J. Campopiano [a] *

[a] School of Chemistry University of Edinburgh, David Brewster Road, Edinburgh, EH9 3FJ.

[b] Diamond Light Source Ltd, Harwell Science & innovation Campus, Didcot, OX11 0DE

Correspondence to: Dominic.Campopiano@ed.ac.uk

Table of Contents

Figure. S1: ***Corneybacterium amycolatum*** **SK46 BioWF sequence** 3

Figure. S2: ***Ca*****BioWF recombinant protein sequence** 4

Figure. S3: Sequence alignment of ***Ca*****BioWF against** ***Bs*****BioW and** ***Aa*****BioW** 5

Figure. S4: N-terminal sequence alignment of the new ***Ca*****BioWF construct against** ***Bs*****BioW and** ***Aa*****BioW shown alongside new** ***Ca*****BioWF sequence** 6

Figure. S5: New ***Ca*****BioWF pET-28a construct with N-terminal extension** 7

Figure. S6: New ***Ca*****BioWF expression tests** 8

Figure. S7: Purification of recombinant ***Ca*****BioWF from** ***E. coli*****.** 9

Figure. S8: ***Ca*****BioW pimeloyl-CoA reaction** 10

Figure. S9: ***Ca*****BioWF kinetic MesG assay** 11

Figure. S10: Sequence alignment of ***Ca*****BioF domain alongside** ***Ec*****BioF** 12

Figure. S11: UV-Vis analysis of full ***Ca*****BioWF reaction** 13

Figure. S12: Analysis of the amino acid substrate scope of the ***Ca*****BioWF fusion.** 14

Figure. S13: Calibration curve of AON formation catalysed by ***Ca*****BioWF** 16

Figure. S14: ***Ca*****BioWF acyl-CoA formation with a variety of di-carboxylic acid lengths** 17

Figure. S15: MS analysis of ***Ca*****BioWF acyl-CoA di-acid reactions** 18

Figure. S16: ***Ca*****BioWF acyl-CoA formation with a variety of mono-carboxylic acid lengths** 19

Figure. S17: MS analysis of ***Ca*****BioWF acyl-CoA mono-acid reactions** 20

Figure. S18: ***Ca*****BioWF F192Y purification** 21

Figure. S19: ***Ca*****BioWF F192Y acyl-CoA reactions** 22

Figure. S20: Full ***Ca*****BioWF reactions with various di and mono-carboxylic acid chain lengths.** 23

Figure. S21: ***Ca*****BioWF unusual carboxylic acid reactions** 24

Figure. S22: pLDDT and pTM scores of the predicted CaBioWF domains 26

Figure. S23: A closer inspection of the predicted ***Ca*****BioW domain** 27

Figure. S24: A closer inspection of the predicted ***Ca*****BioF domain** 28

Figure. S25: Visualisation of the CaBioWF simulation 29

Figure. S26: A summary of the ***Ca*****BioWF MDS** 30

Figure. S27: Fluctuation analysis of the simulated CaBioWF complex 31

Figure. S28: The ***Ca*****BioWF complex sampled midway (t = 5 ns) through the MDS** 32

Figure. S29: A predicted molecular "tunnel" (dashed line) between the ***Ca*****BioW and** ***Ca*****BioF domains.** 33

Supplementary Figures:

ATGCGCTCGTCCGCTGATTACTGTCACATCTCGGGTGCTGAACGCCTGGCCCCGGCTACCGAACTGCCGCAAC
TGGCATCCGCAATGACCAGCCGTGCCCTGCATCACGATAAAGGCCGCCCGGACACCATCCATATCACGGTTGA
TAAAATCGAAGAATCCACCATCTCAACGGTCCCGGCGCTGACCCCGTTTCTGGAAAGCAACAGCTCTCCGGGC
GATGCTCGCAAACTGATTGCGCAGCGTCTGCACGCGGCCGGTATTCAAGCAGCTGATATCGCGGCCGAAATG
GCCTATAGCCTGACCGGCCTGCGTGGTGCAGCTCTGATCGATAGTTCCTCAGGTGAACGTCTGGACCCGAATC
CGGCCCGCGGTGTTCGTGTCAGCACCTTTGATGCTATTTCGCATCCGAGCAAAGATTGCGCAAAAGACCATTT
CCACGAAGCCCTGATCCTGGCATCGAAAGTTCACAGCGCCCCGGGCATTGTCGCAGAAATCTGTCTGTCTGAT
GACCCGTTTTATACCCGTGGCTACCTGGCGCTGGATGGCTTTTTCCATCGCATTCCGAACATCAAAGACCACGG
CAGTACCCTGGGTACGCGTATTTTCATCGTGGAACCGGATACCGACATTCCGGAACTGATCGATTATCTGGAA
AATACGCCGGTTTACATTGAACTGCCGCCGGATGCCTCGAGCTCTACCGACACCACGGGTCTGAGTTCCGATC
TGAGCGCAATTGCCGCACAGCGTAATACCGCCTGGGCAGGTGCAGGTCTGACCCGCACGCTGCGTACCTTTG
AAACGGCGCAACTGCCGCATAGTCGCATTGATGGCGCCGACTATCTGCTGTTCTCATCGAGCGATTACCTGGG
TCTGTCCACCCATCCGGAACTGGTGTCAGCAGCTACCGCGGCCATTGGCCACTTTGGCACGGGTTCTGGCGGT
AGTCGTCTGACCACGGGTACCAGCATCCATTCAGCTCTGGAATCGGAACTGGCGCAGTTTTTCGGCTTTGATG
ACGCTGTCCTGTTCGCGACGGGTTATCAAGCGAACCATAGCACCATTGCAGCTATCGCTACGGCGGATGTTGA
AATTTTTAGCGACGCGGCCAACCACGCCAGCATTATCGATGGTTGCCGCAATGCCCGTGCAAAAGTGACCGTT
TTCCCGCATGCGGATTACCAGACCCTGGACCGTCTGCTGGCCACGTCTAGTGCACGCCACAAACTGGTGATTT
CCGATTCAGTGTTTAGCATGAGCGGCGAAGTGATTGATGGTCCGGCCCTGGAACGTACCTGCCGTCGCCGTAA
TGCCTGGCTGATGCTGGATGACGCACATGGCGTCGGTGTGATTGGCGAACAGGGCCGTGGTACCGCAGCTCA
CCTGGATATTCGTCCGGACATTGTGGTTGGTACCGCATCCAAAGCACTGGGTGTCGAAGGCGGTTATGTGCTG
TGTTCAGCACCGGTTGGTGAACTGCTGCGCAACCAGGCGCGTTCTTTTGTGTACTCTACCAGTATGAATCCGG
GCAGTGTTGCGGCCATTCGTGCAGCTCTGAAACAGCTGGAAGTGGGTGATGTCGTGAAACGCCTGCAACGTA
ATATTGCTCGTGTTCTGTCCCTGGTCGGCGCCCAGTCTGATCCGGCTAGTGCGATTATCCCGCTGCCGGTTGGT
GATGAAACCGAAGCCATGGACATTTCTGCTCAACTGGCGGAACTGGGCGTCTTCATTCCGGCCATCCGTTATC
CGACCGTGCCGCGTGGTGAAGCAATGCTGCGTCTGACCATCACGGCACTGCACACCGATGCCGACATTGACC
AACTGGAACTGGCGCTGCGTAACACGGGCCTGCTG

Figure. S1: *Corneybacterium amycolatum* SK46 BioWF sequence
Original sequence of the *Corneybacterium amycolatum* SK46 BioWF (Uniprot: E2MUP3).

MGSSHHHHHHSSGLVPRGSH**M**RSSADYCHISGAERLAPATELPQLASAMTSRALHHDKGRPDTIHITVDKIEESTIS
TVPALTPFLESNSSPGDARKLIAQRLHAAGIQAADIAAEMAYSLTGLRGAALIDSSSGERLDPNPARGVRVSTFDAIS
HPSKDCAKDHFHEALILASKVHSAPGIVAEICLSDDPFYTRGYLALDGFFHRIPNIKDHGSTLGTRIFIVEPDTDIPELID
YLENTPVYIELPPDASSSTDTTGLSSDLSAIAAQRNTAWAGAGLTRTLRTFETAQLPHSRIDGADYLLFSSSDYLGLST
HPELVSAATAAIGHFGTGSGGSRLTTGTSIHSALESELAQFFGFDDAVLFATGYQANHSTIAAIATADVEIFSDAANH
ASIIDGCRNARAKVTVFPHADYQTLDRLLATSSARHKLVISDSVFSMSGEVIDGPALERTCRRRNAWLMLDDAHGV
GVIGEQGRGTAAHLDIRPDIVVGTASKALGVEGGYVLCSAPVGELLRNQARSFVYSTSMNPGSVAAIRAALKQLEV
GDVVKRLQRNIARVLSLVGAQSDPASAIIPLPVGDETEAMDISAQLAELGVFIPAIRYPTVPRGEAMLRLTITALHTD
ADIDQLELALRNTGLL

Figure. S2: *Ca*BioWF recombinant protein sequence
The recombinant protein sequence of the original pET28a construct of original *Ca*BioWF sequence of *Corneybacterium amycolatum* SK46 BioWF (starting Met shown in bold) with an N-terminal HisTag.

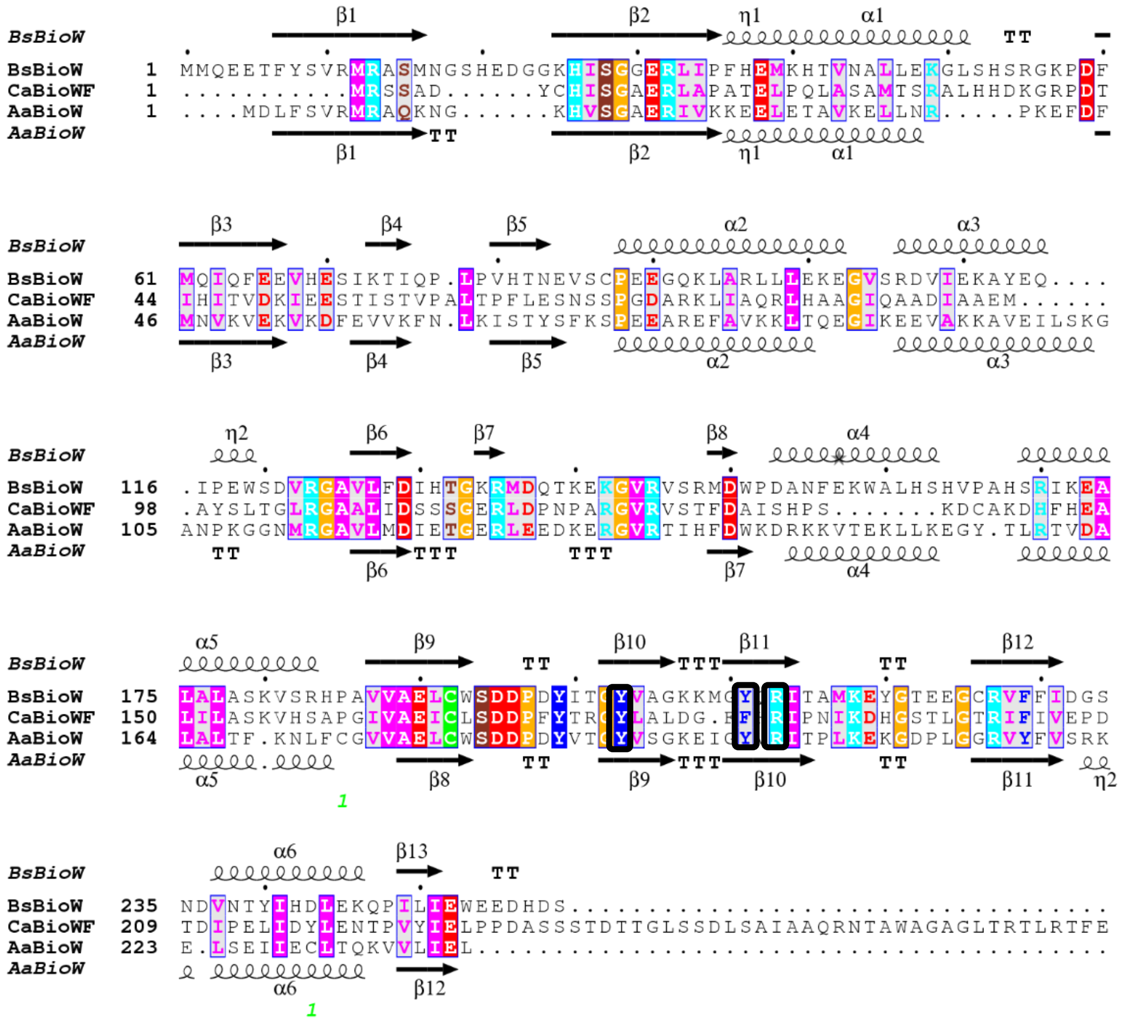

Figure. S3: Sequence alignment of *Ca*BioWF against *Bs*BioW and *Aa*BioW

Sequence alignment of the Genbank predicted BioW domain of *Ca*BioWF against its closest homologues *Bs*BioW (PDB: **5FLL**, Uniprot: P53559) and *Aa*BioW (PDB: **5TV6**, Uniprot: O67575). The predicted active site residues are highlighted.

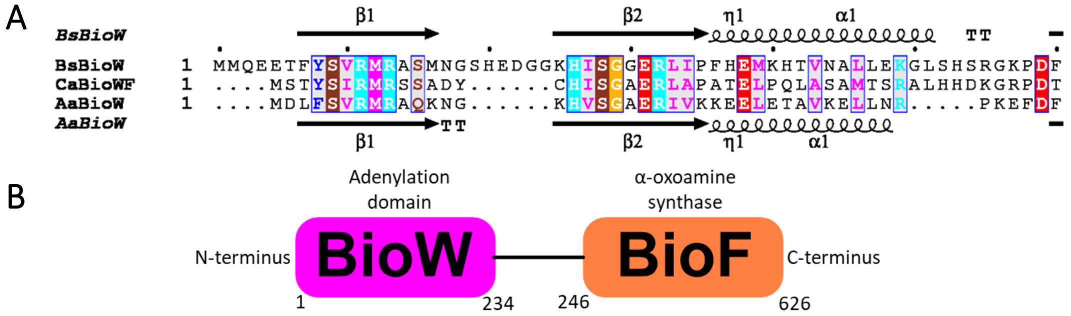

C

```
MSTYSIRMRS SADYCHISGA ERLAPATELP QLASAMTSRA LHHDKGRPDT IHITVDKIEE 60
STISTVPALT PFLESNSSPG DARKLIAQRL HAAGIQAADI AAEMAYSLTG LRGAALIDSS 120
SGERLDPNPA RGVRVSTFDA ISHPSKDCAK DHFHEALILA SKVHSAPGIV AEICLSDDPF 180
YTRGYLALDG FFHRIPNIKD HGSTLGTRIF IVEPDTDIPE LIDYLENTPV YIELPPDASS 240
STDTTGLSSD LSAIAAQRNT AWAGAGLTRT LRTFETAQLP HSRIDGADYL LFSSSDYLGL 300
STHPELVSAA TAAIGHFGTG SGGSRLTTGT SIHSALESEL AQFFGFDDAV LFATGYQANH 360
STIAAIATAD VEIFSDAANH ASIIDGCRNA RAKVTVFPHA DYQTLDRLLA TSSARHKLVI 420
SDSVFSMSGE VIDGPALERT CRRRNAWLML DDAHGVGVIG EQGRGTAAHL DIRPDIVVGT 480
ASKALGVEGG YVLCSAPVGE LLRNQARSFV YSTSMNPGSV AAIRAALKQL EVGDVVKRLQ 540
RNIARVLSLV GAQSDPASAI IPLPVGDETE AMDISAQLAE LGVFIPAIRY PTVPRGEAML 600
RLTITALHTD ADIDQLELAL RNTGLL 626
```

Figure. S4: N-terminal sequence alignment of the new *Ca*BioWF construct against *Bs*BioW and *Aa*BioW shown alongside new *Ca*BioWF sequence

A) New N-terminal sequence after addition of 7 amino acids (MSTYSIR) to the N-terminus of *Ca*BioWF (Uniprot: E2MUP3), aligned with *Bs*BioW (*Bacillus subtilis*, Uniprot: P53559) and *Aa*BioW (*Aquifex aeolicus*, Uniprot: O67575). **B)** *Ca*BioWF schematic of the didomain protein showing the BioW domain (pink, residues 1-227) and BioF domain (orange, residues 239-619) along with the **C)** new *Ca*BioWF recombinant protein sequence with the BioW ANL domain (pink), the BioF AON domain (orange) and the linker region (black). The predicted ANL active site residues (Tyr181, Phe192 and Arg194) and key BioF residues including the key PLP binding lysine (Arg271, Ser295, His379, Lys483) are shown in a darker shade and underlined.

MGSTYSIR**M**RSSADYCHISGAERLAPATELPQLASAMTSRALHHDKGRPDTIHITVDKIEESTISTVPALTPFLESNSS

PGDARKLIAQRLHAAGIQAADIAAEMAYSLTGLRGAALIDSSSGERLDPNPARGVRVSTFDAISHPSKDCAKDHFHE

ALILASKVHSAPGIVAEICLSDDPFYTRGYLALDGFFHRIPNIKDHGSTLGTRIFIVEPDTDIPELIDYLENTPVYIELPPD

ASSSTDTTGLSSDLSAIAAQRNTAWAGAGLTRTLRTFETAQLPHSRIDGADYLLFSSSDYLGLSTHPELVSAATAAIG

HFGTGSGGSRLTTGTSIHSALESELAQFFGFDDAVLFATGYQANHSTIAAIATADVEIFSDAANHASIIDGCRNARAK

VTVFPHADYQTLDRLLATSSARHKLVISDSVFSMSGEVIDGPALERTCRRRNAWLMLDDAHGVGVIGEQGRGTAA

HLDIRPDIVVGTASKALGVEGGYVLCSAPVGELLRNQARSFVYSTSMNPGSVAAIRAALKQLEVGDVVKRLQRNIAR

VLSLVGAQSDPASAIIPLPVGDETEAMDISAQLAELGVFIPAIRYPTVPRGEAMLRLTITALHTDADIDQLELALRNTG

LLGSENLYFQ**GLEHHHHHH**

Figure. S5: New *Ca*BioWF pET-28a construct with N-terminal extension

Final *Corneybacterium amycolatum* SK46 (Uniprot: E2MUP3) *Ca*BioWF recombinant protein sequence in a pET-28a plasmid with a C-terminal TEV cleavable HisTag shown in bold. New and old starting Met and also shown in bold, extension added in yellow.

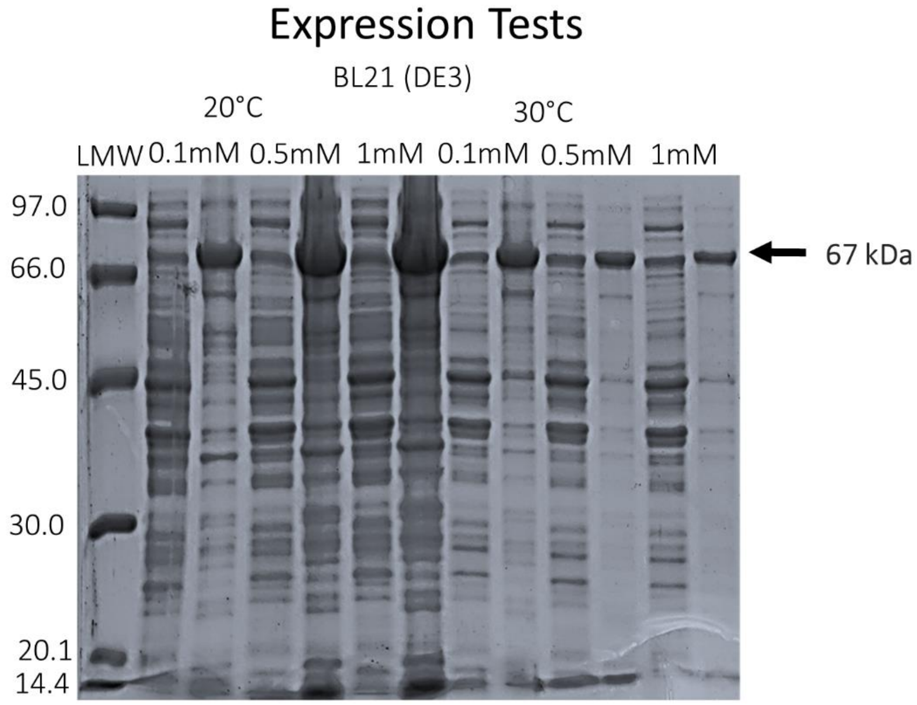

Figure. S6: New *Ca*BioWF expression tests

Expression tests of new pET-28a/*Ca*BioWF construct in BL21 (DE3) cells, with expression tested under the two temperatures of 20 °C o/n and 30 °C for 5 hours at IPTG concentrations of 0.1 mM, 0.5 mM and 1 mM.

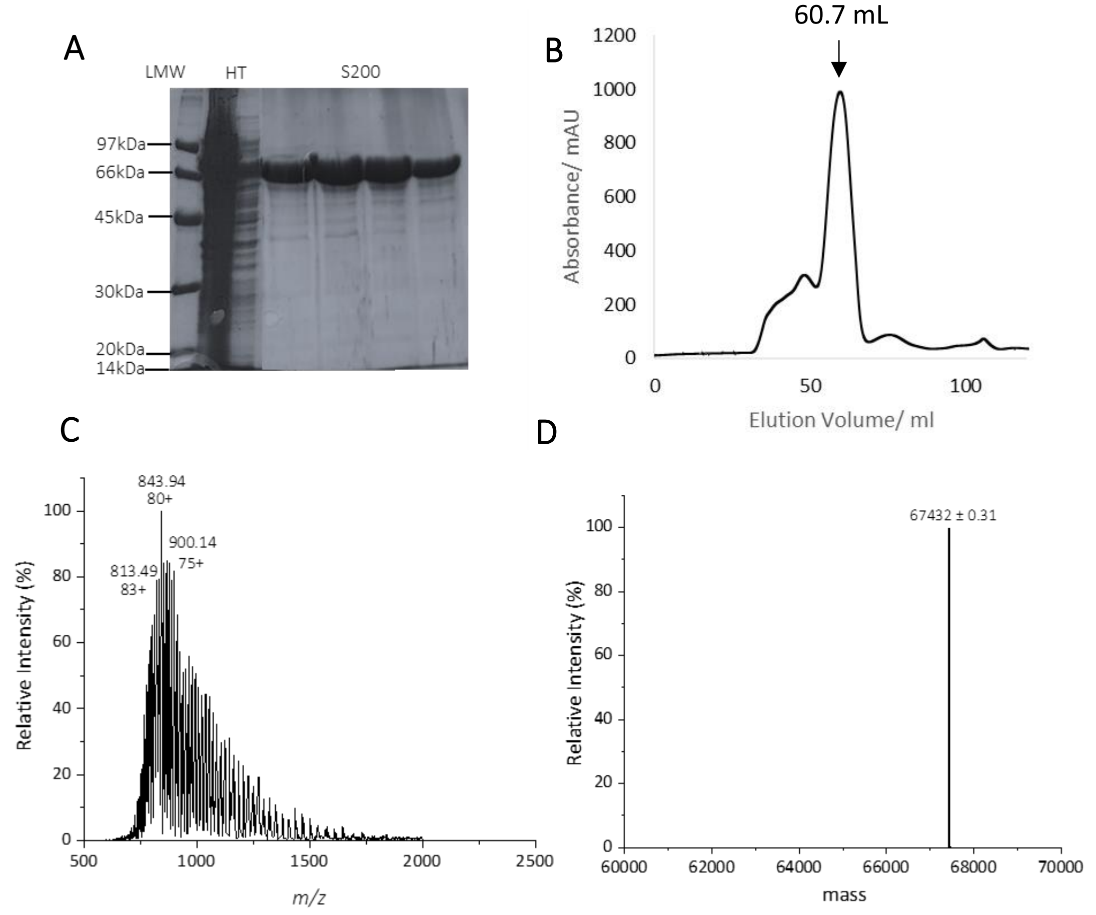

Figure. S7: Purification of recombinant *Ca*BioWF from *E. coli*.

Purification of the new *Ca*BioWF construct using A) SDS-PAGE analysis with LMW marker shown alongside eluted HT fractions and Superdex S200 fractions corresponding to the eluted S200 peak at 60.7 mL, B) S200 SEC chromatogram monitored at 280 nm with an elution volume of 60.7 mL and C) LC ESI-MS analysis showing the charge states and D) deconvoluted mass of the obtained protein at 67432.45 ± 0.31 Da.

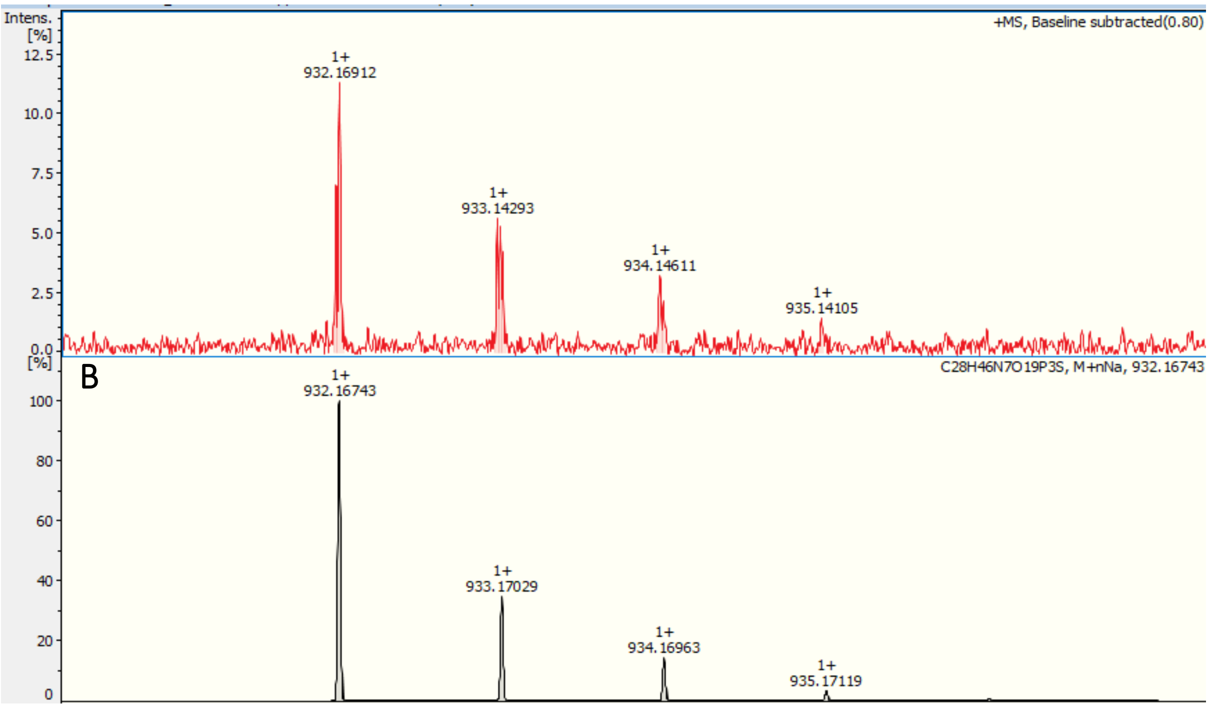

Figure. S8: *Ca*BioW pimeloyl-CoA reaction

FT-ICR MS analysis of the pimeloyl-CoA intermediate formed by the *Ca*BioW domain shown by the presence of **A)** the ion m/z = 932.1674 aligned with **B)** the predicted mass of 932.1674 ($[M + Na]^+$, $C_{28}H_{46}N_7O_{19}P_3S$).

A

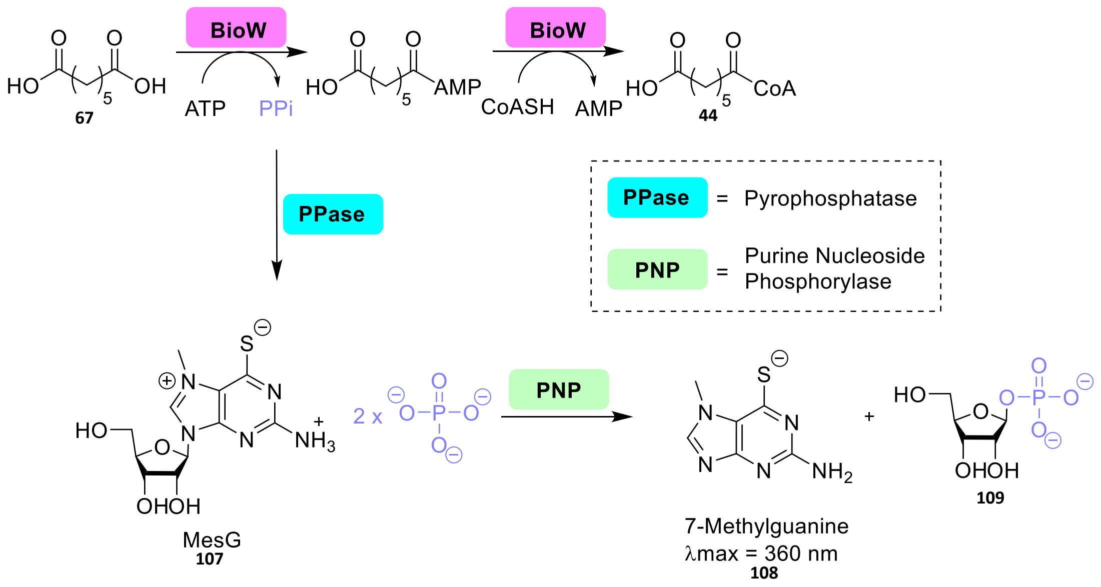

B

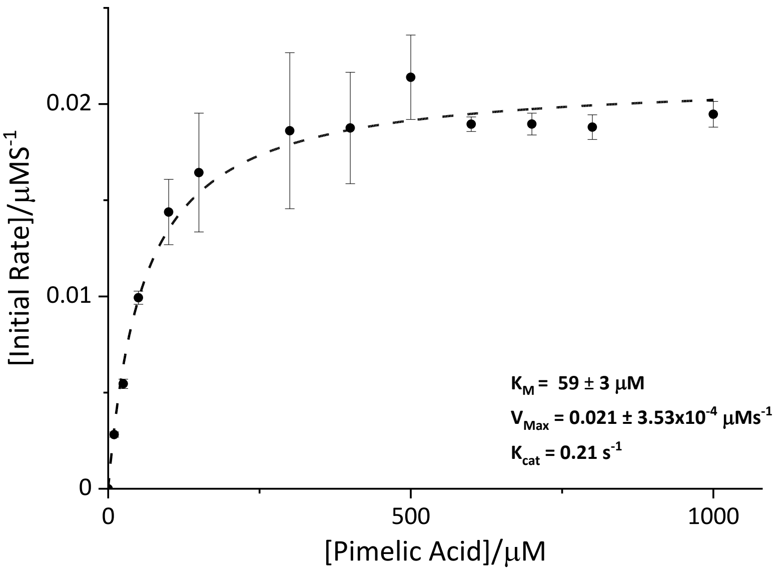

Figure. S9: ***Ca*****BioWF kinetic MesG assay**

Schematic of the coupled pyrophosphate production MesG assay. The pimelic acid is activated by the ANL enzyme *Bs*BioW using ATP, releasing PPi. The PPi is broken into two molecules of Pi by PPase. The Pi is then utilised with MesG by PNP to form 7-methylguanine, which absorbs at 360 nm. Michaelis-Menten analysis of the purified *Ca*BioWF using the coupled MesG assay with a calculated K_M value of 59 ± 3 μM.

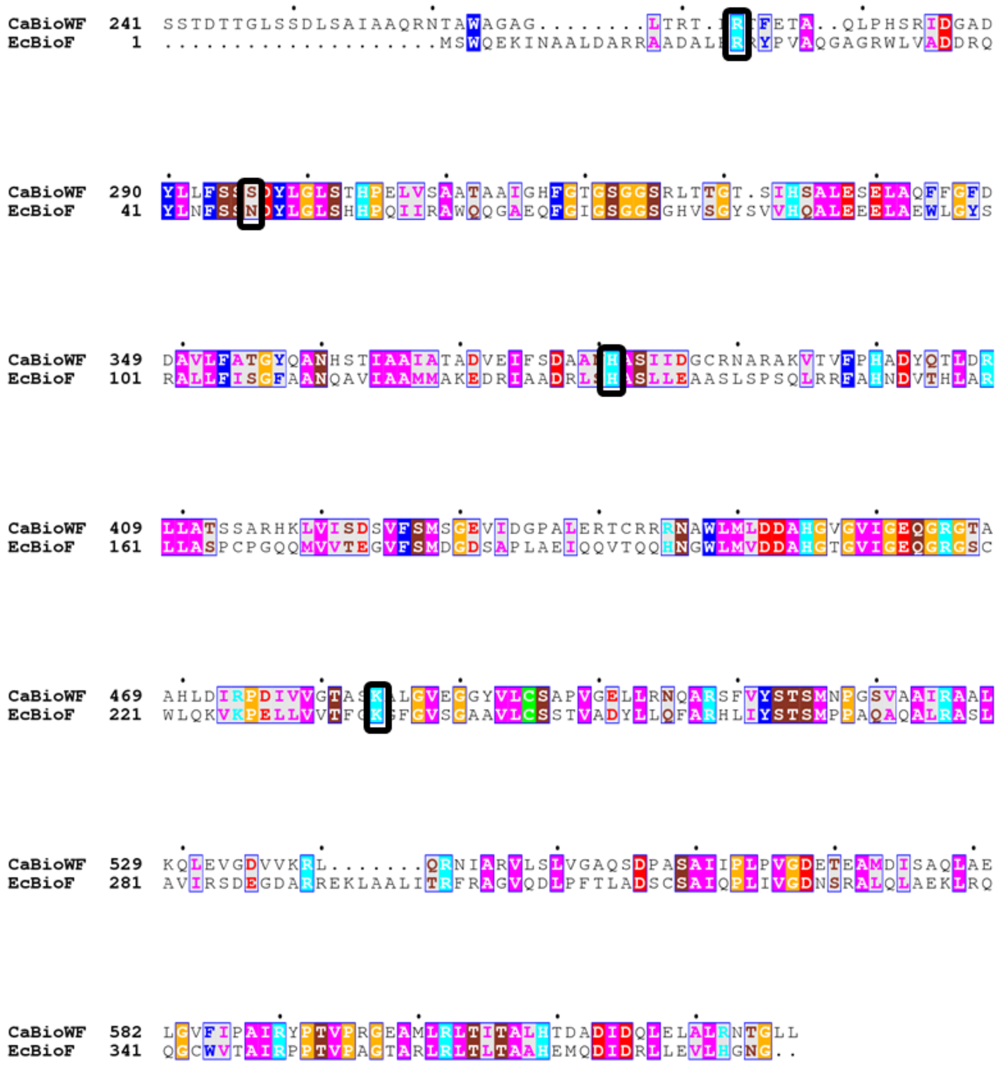

Figure. S10: Sequence alignment of *Ca*BioF domain alongside *Ec*BioF

Sequence alignment of the *Ca*BioF domain of *Ca*BioWF against its closest homologues *Ec*BioF (PDB: 1DJ9, Uniprot: P12998) with key active site residues Arg21, Asn47, His133 and Lys236 (*Ec*BioF numbering).

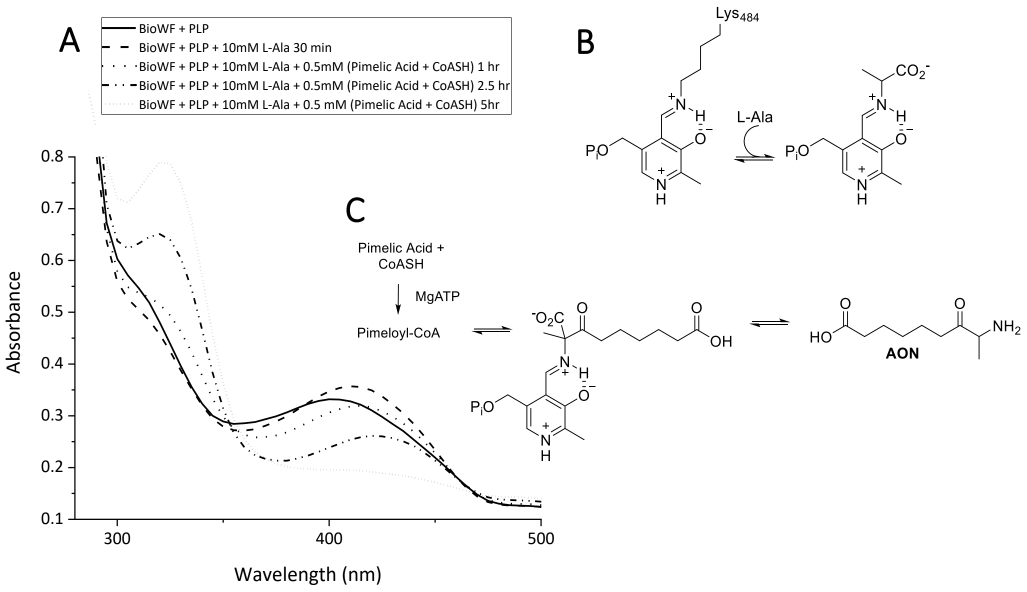

Figure. S11: UV-Vis analysis of full *Ca*BioWF reaction

A) UV-Vis spectroscopy scan of *Ca*BioWF PLP-binding, monitoring the changes after the addition of L-Ala (10 mM) followed by pimelic acid and CoASH in the presence of $MgCl_2$ and ATP, forming pimeloyl-CoA by the *Ca*BioW domain. This intermediate is used by the *Ca*BioF domain, monitoring changes in the absorbance for AON formation. Schematic of B) conversion from internal to external aldimine and C) condensation of pimeloyl-CoA and L-Ala by *Ca*BioF domain leading to absorbance changes also shown.

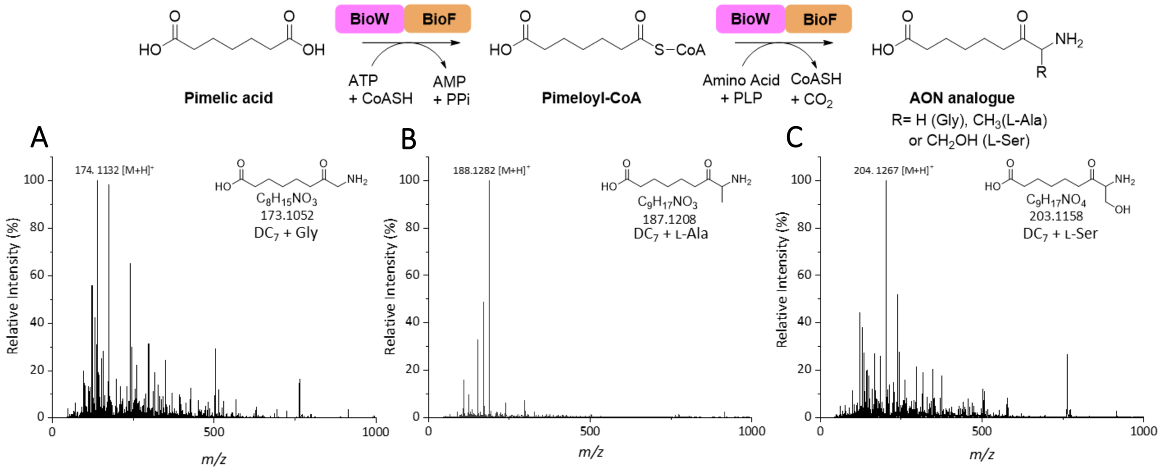

Figure. S12: Analysis of the amino acid substrate scope of the *Ca*BioWF fusion.

LC ESI-MS analysis of the full *Ca*BioWF reaction upon incubation with pimelic acid, MgATP and CoASH leading to first the production of the BioW-catalysed pimeloyl-CoA intermediate and then the BioF-catalysed C-C bond forming reaction with either **A)** Gly, **B)** L-Ala or **C)** L-Ser, each leading to the formation of the corresponding aminoketone AON product.

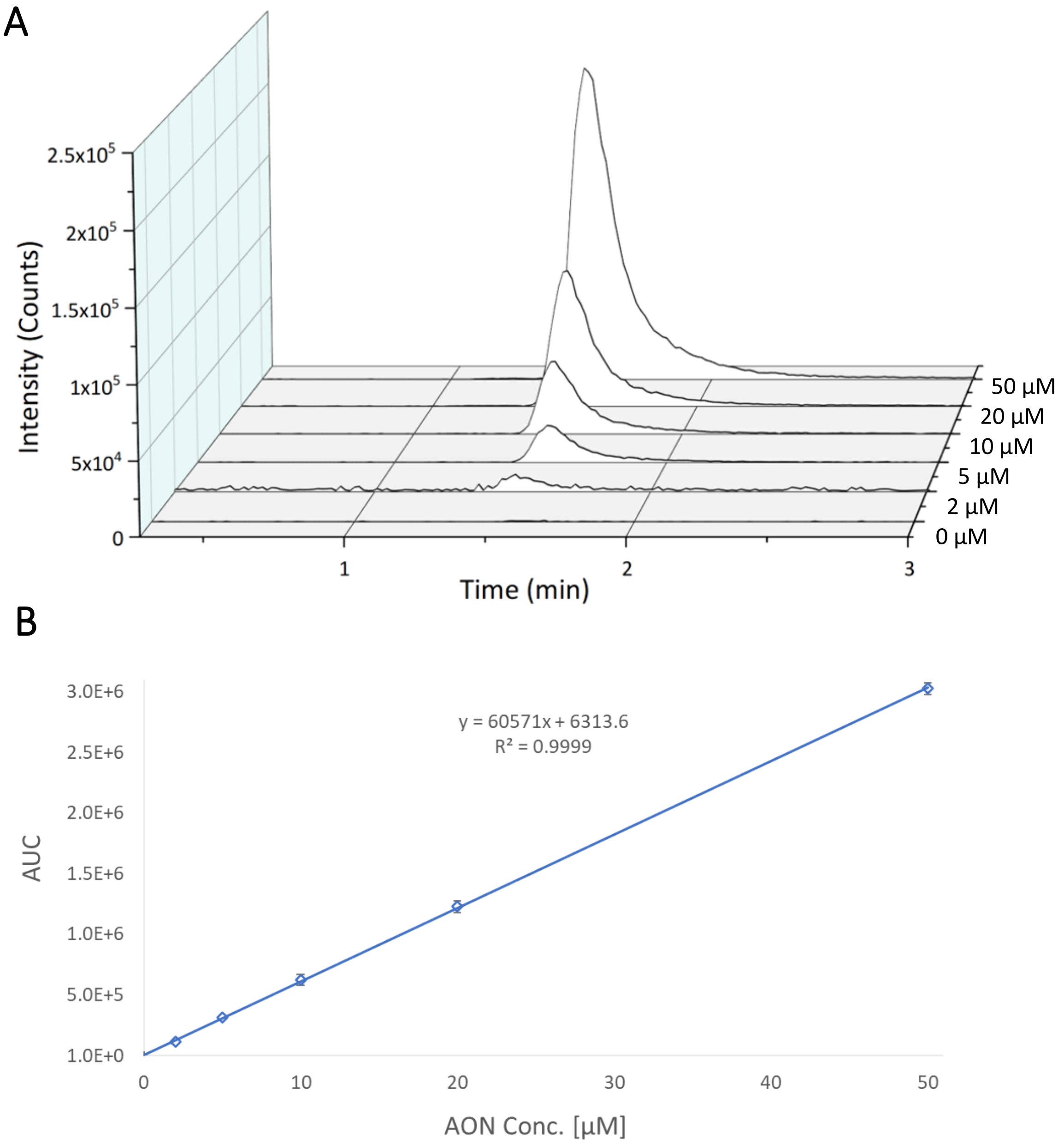
A
2.5x10^5
2x10^5
1.5x10^5
1x10^5
5x10^4
0
Intensity (Counts)
50 µM
20 µM
10 µM
5 µM
2 µM
0 µM
1
2
3
Time (min)
B
3.0E+6
2.5E+6
2.0E+6
1.5E+6
1.0E+6
5.0E+5
1.0E+0
AUC
y = 60571x + 6313.6
R² = 0.9999
0
10
20
30
40
50
AON Conc. [µM]

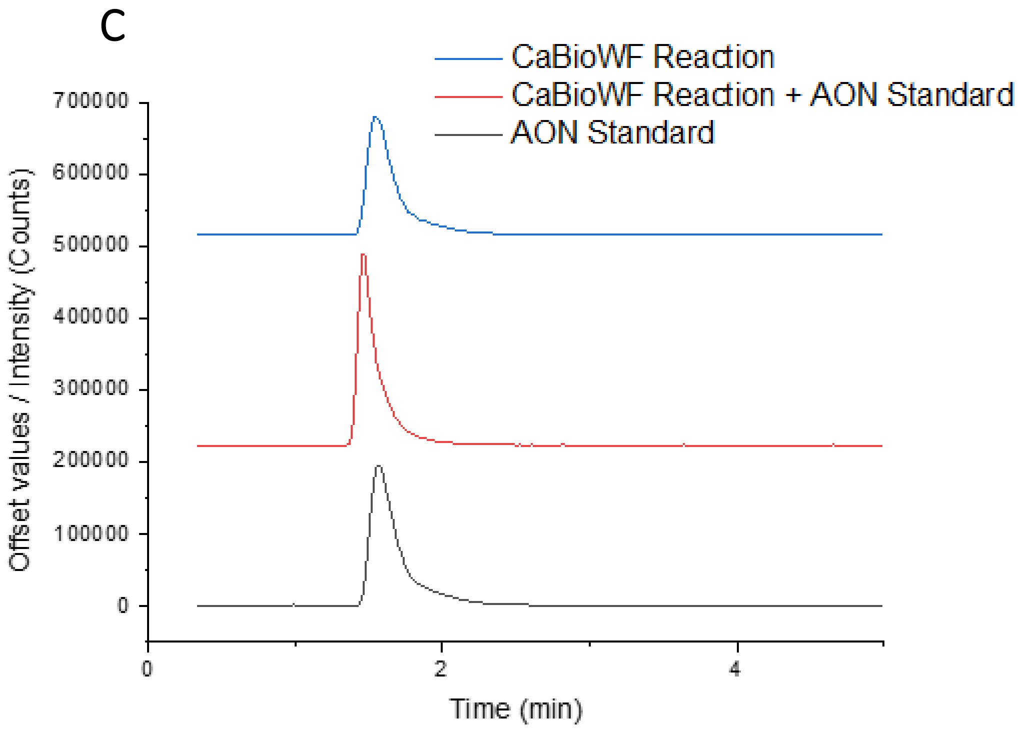

Figure. S13: Calibration curve of AON formation catalysed by *Ca*BioWF

Calibration curve of AON. **A)** Extracted Ion Chromatograms (EICs) of calibration solutions (0-50 µM) of commercial AON $[M+H]^+$ *m/z* = 188.1282 + 0.01 Da. **B)** Calibration curve of the area under the curve (AUC) of the repeats of each calibration solution. **C)** The EICs of a standard solution of AON, the *Ca*BioWF reaction and a mixture of both the *Ca*BioWF reaction and the AON standard, $[M+H]^+$ *m/z* = 188.1282, showing only one peak with the expected mass with the same RT. The reaction was performed using *Ca*BioWF (5 µM), TCEP (0.2 mM), ATP (1 mM), CoASH (0.5 mM), pimelic Acid (1.5 mM) and L-serine (1.5 mM) in buffer (Tris.HCl (25 mM, pH 8), NaCl (50 mM), $MgCl_2$ (5 mM)) in 10 mL final volume, heated at 30 °C for 5 hrs with 180 rpm agitation. The reaction was quenched using 1.7 % TFA (60 µL per 1 mL of reaction) and centrifuged at 17000 xg for 10 minutes. The supernatant was diluted (1:20, 1:50) for LCMS analysis using the same buffer to complete the volume.

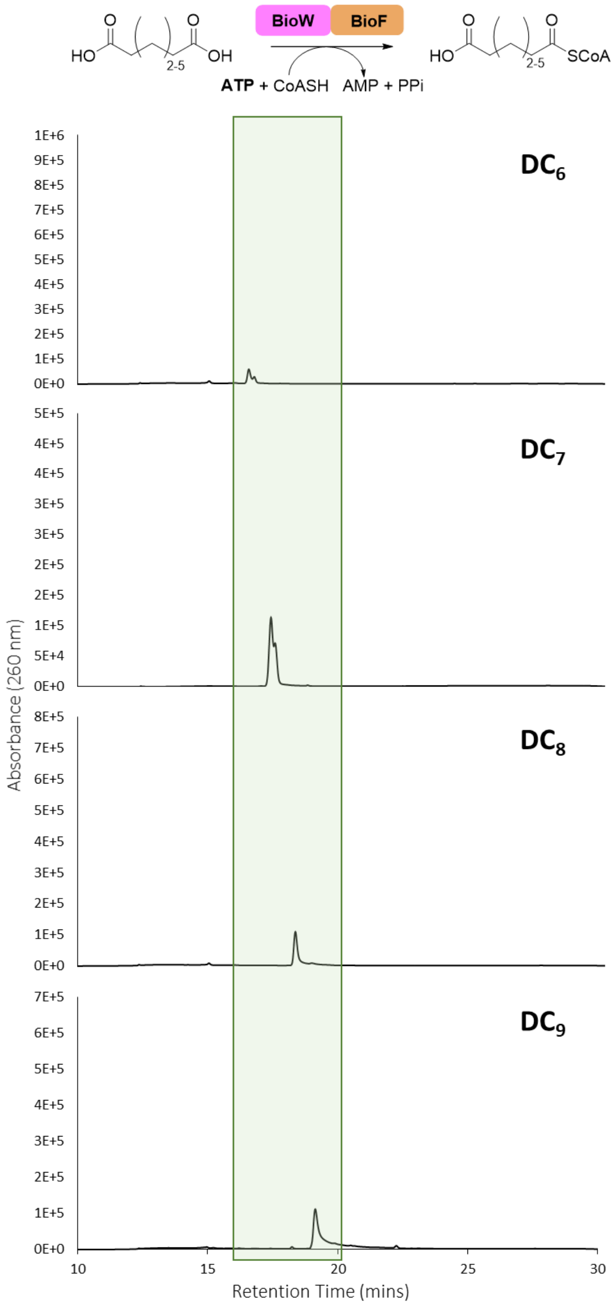

Figure. S14: *Ca*BioWF acyl-CoA formation with a variety of di-carboxylic acid lengths

Demonstration of the substrate promiscuity of the *Ca*BioW domain of *Ca*BioWF fusion. The HPLC assay results (from 10 – 30 mins shown) for *Ca*BioWF enzymatic reactions with DC_6-DC_9 di-acids (schematic shown above the chromatograms) with CoASH leading to the formation of the corresponding acyl-CoA product: DC_6 (16.5 min), DC_7 (17.3 min) DC_8 (18.3 min) and DC_9 (19.1 min).

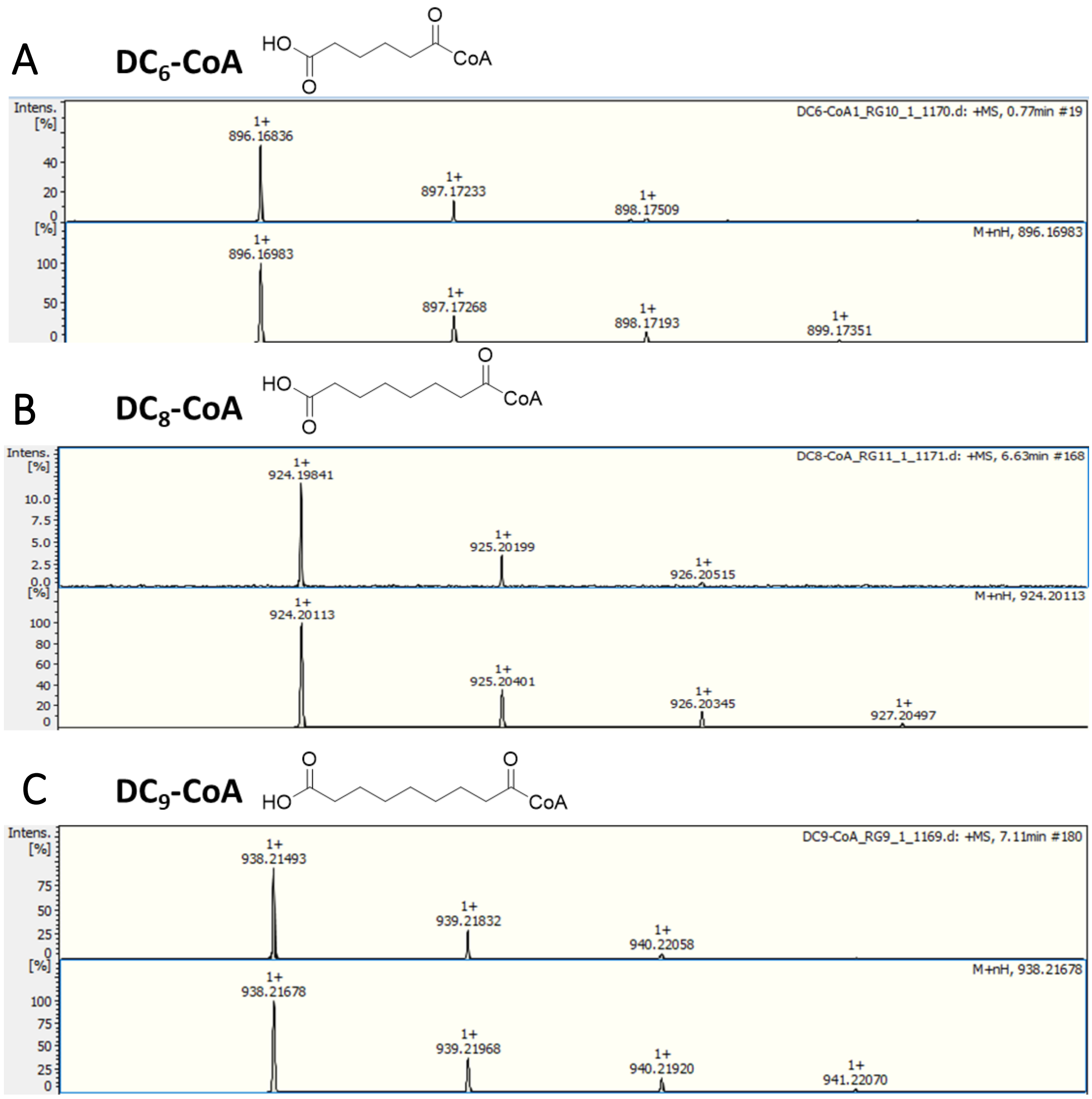

Figure. S15: MS analysis of *Ca*BioWF acyl-CoA di-acid reactions

FT ICR-MS analysis of the *Ca*BioWF reactions of the *Ca*BioW domain upon incubation of the enzyme with varying acyl chains lengths (DC_6-DC_8) and CoASH (DC_7 shown in Fig.S8) with **A)** DC_6-CoA **B)** DC_8-CoA and **C)** DC_9-CoA.

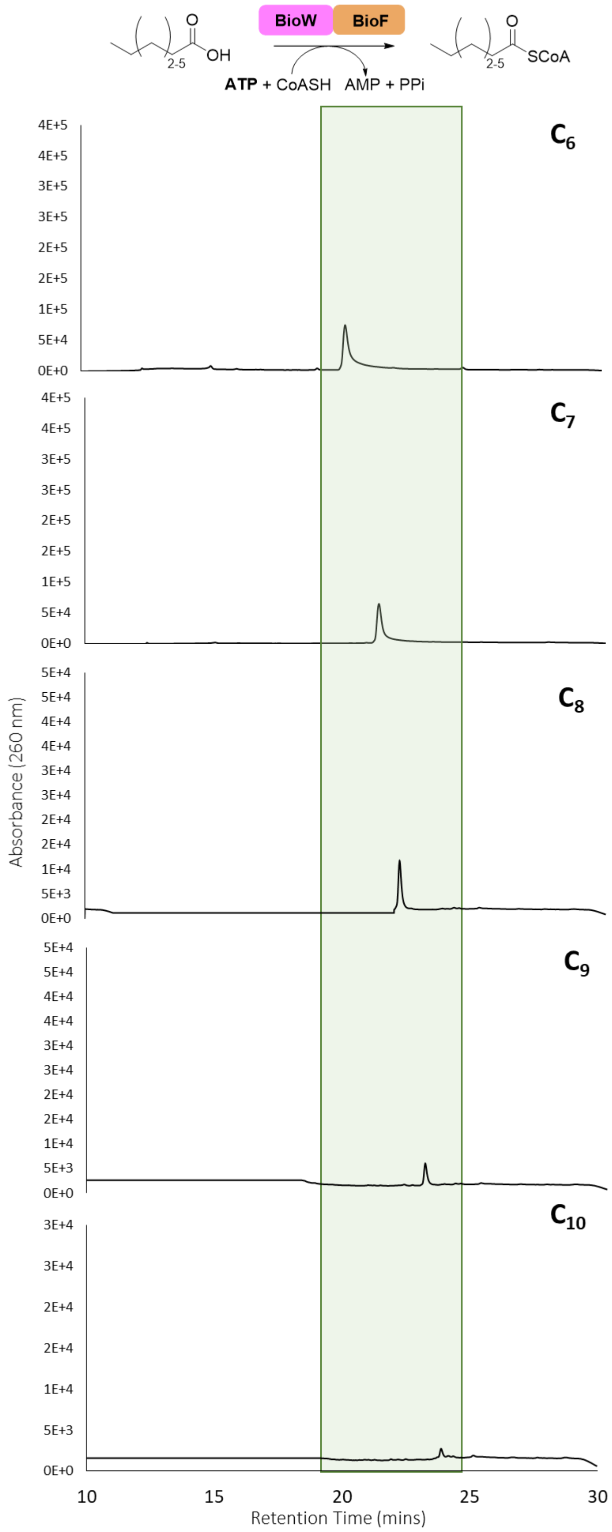

Figure. S16: *Ca*BioWF acyl-CoA formation with a variety of mono-carboxylic acid lengths

Mono-acid substrate promiscuity of the *Ca*BioW domain of the *Ca*BioWF fusion. HPLC assay results for *Ca*BioWF enzymatic reactions with C_6-C_{10} mono-acids (schematic shown above the chromatograms) with CoASH leading to the formation of the corresponding acyl-CoA product (green): C_6 (20.1 mins), C_7 (21.2 mins), C_8 (22.1 min), C_9 (23.4 min) and C_{10} (24.0 min).

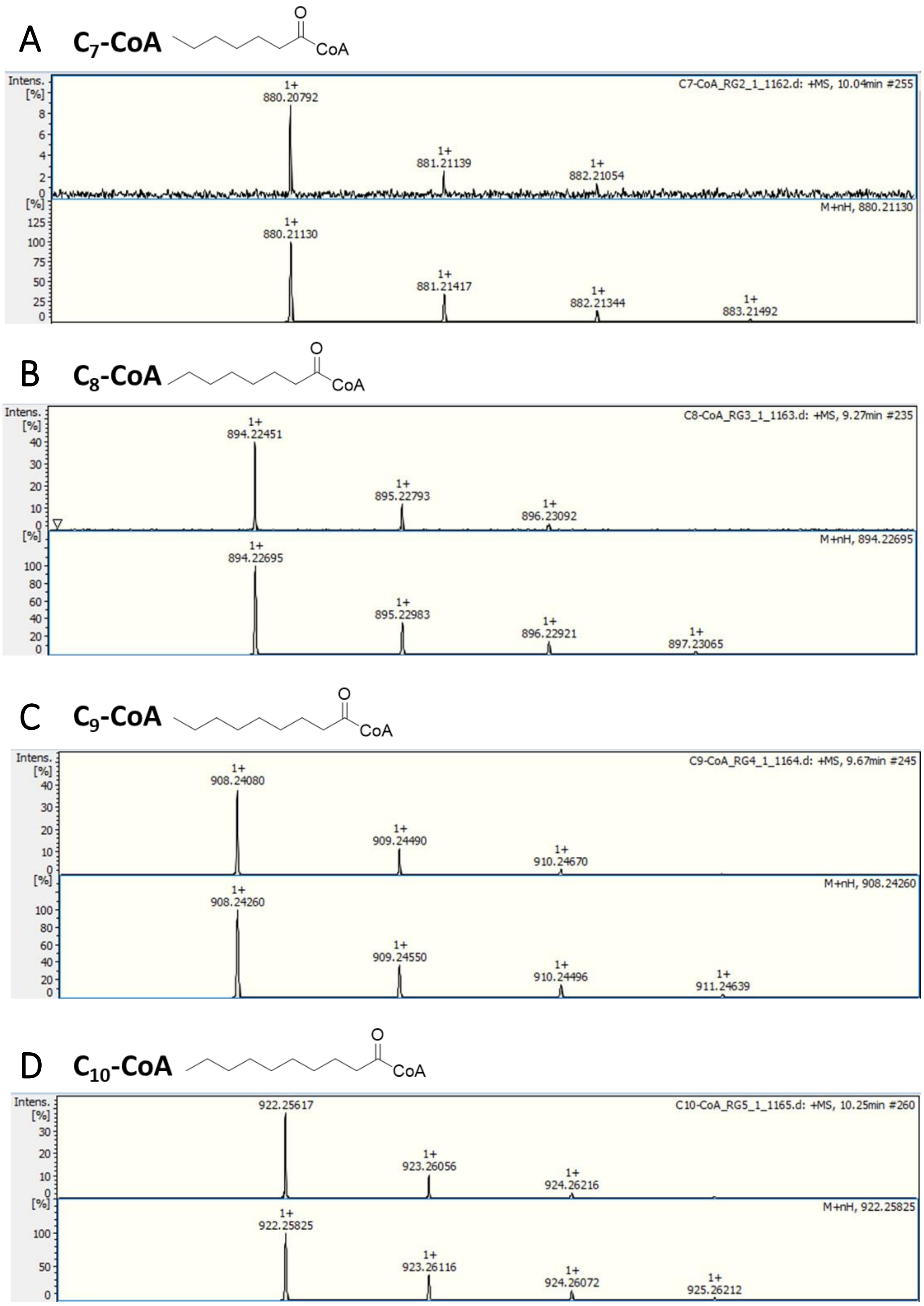

Figure. S17: MS analysis of *Ca*BioWF acyl-CoA mono-acid reactions

FT ICR-MS analysis of the *Ca*BioWF reactions of the *Ca*BioW domain upon incubation of the enzyme with varying acyl chains lengths (C_6-C_{10}) and CoASH (C_6 was not observed) with **A)** C_7-CoA **B)** C_8-CoA, **C)** DC_9-CoA and **D)** C_{10}-CoA.

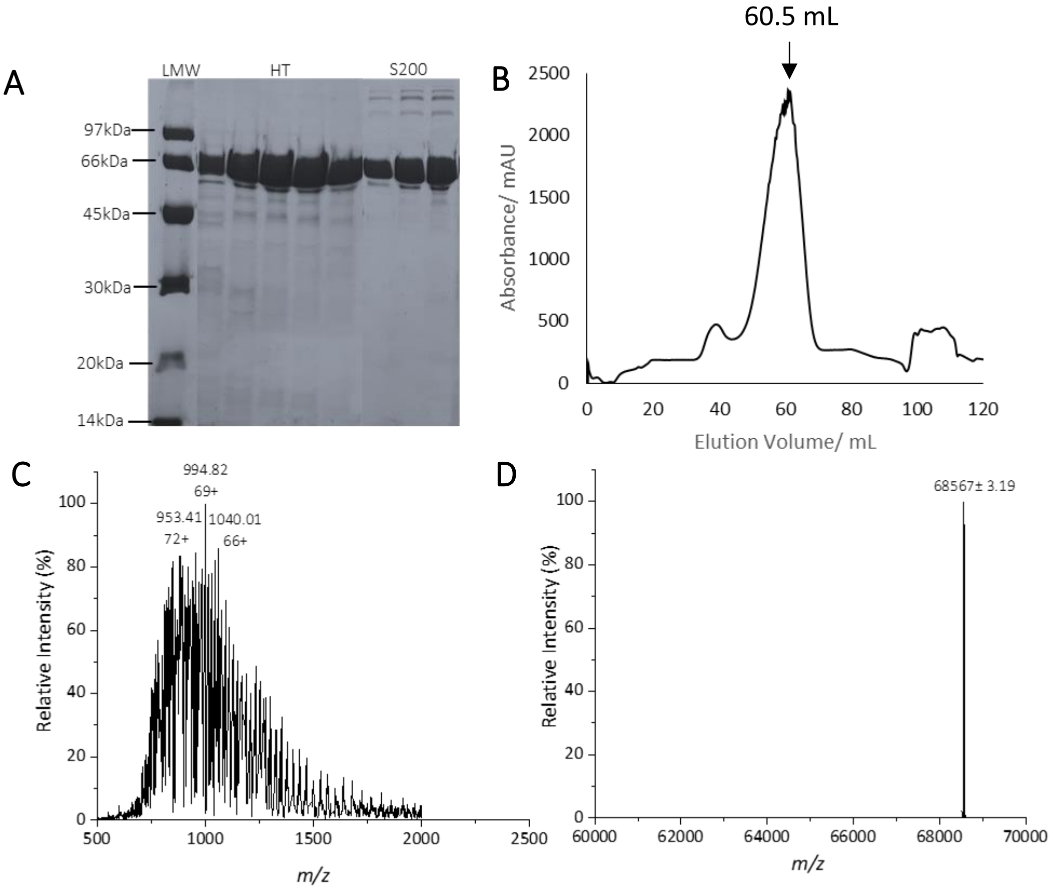

Figure. S18: *Ca*BioWF F192Y purification

Purification of the *Ca*BioWF F192Y mutant using **A)** SDS-PAGE analysis with LMW marker, eluted HT fractions and Superdex S200 fractions corresponding to the peak at 60.5 mL, **B)** S200 SEC chromatogram monitored at 280 nm with an elution volume of 60.5 mL and **C)** LC-ESI MS analysis showing protein charge states of the **D)** obtained protein with a deconvoluted mass of 68567 ± 3.19 Da.

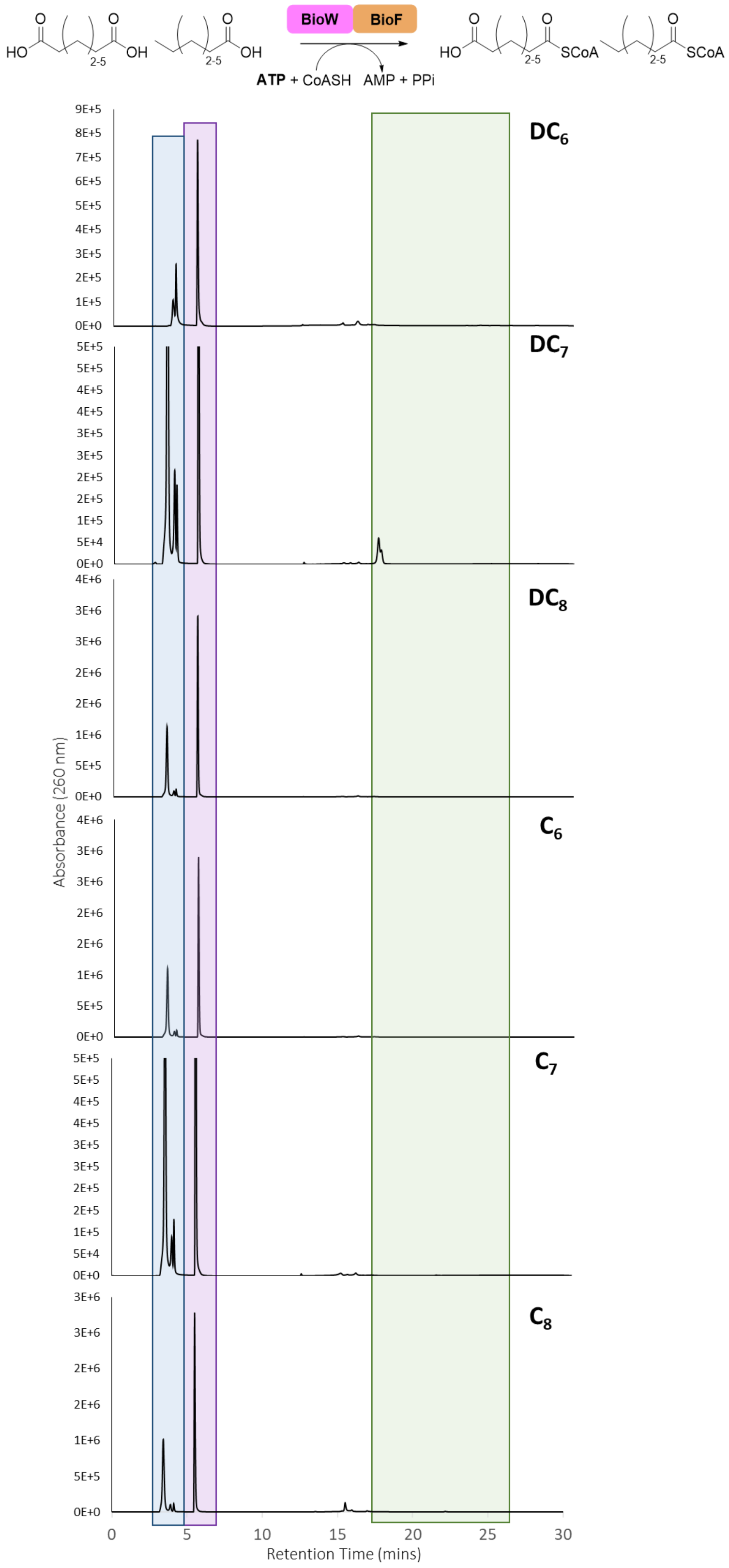

Figure. S19: ***Ca*****BioWF F192Y acyl-CoA reactions**

HPLC assay results for *Ca*BioWF F192Y mutant with DC_6 – DC_8 and C_6- C_8 (schematic shown above the chromatograms) with CoASH leading to the formation of the corresponding acyl-CoA product (green) for DC_7-CoA (17.3 mins) but no acyl-CoA product for the remaining reactions (green) is visible. ATP (purple) and released AMP (blue) are also shown.

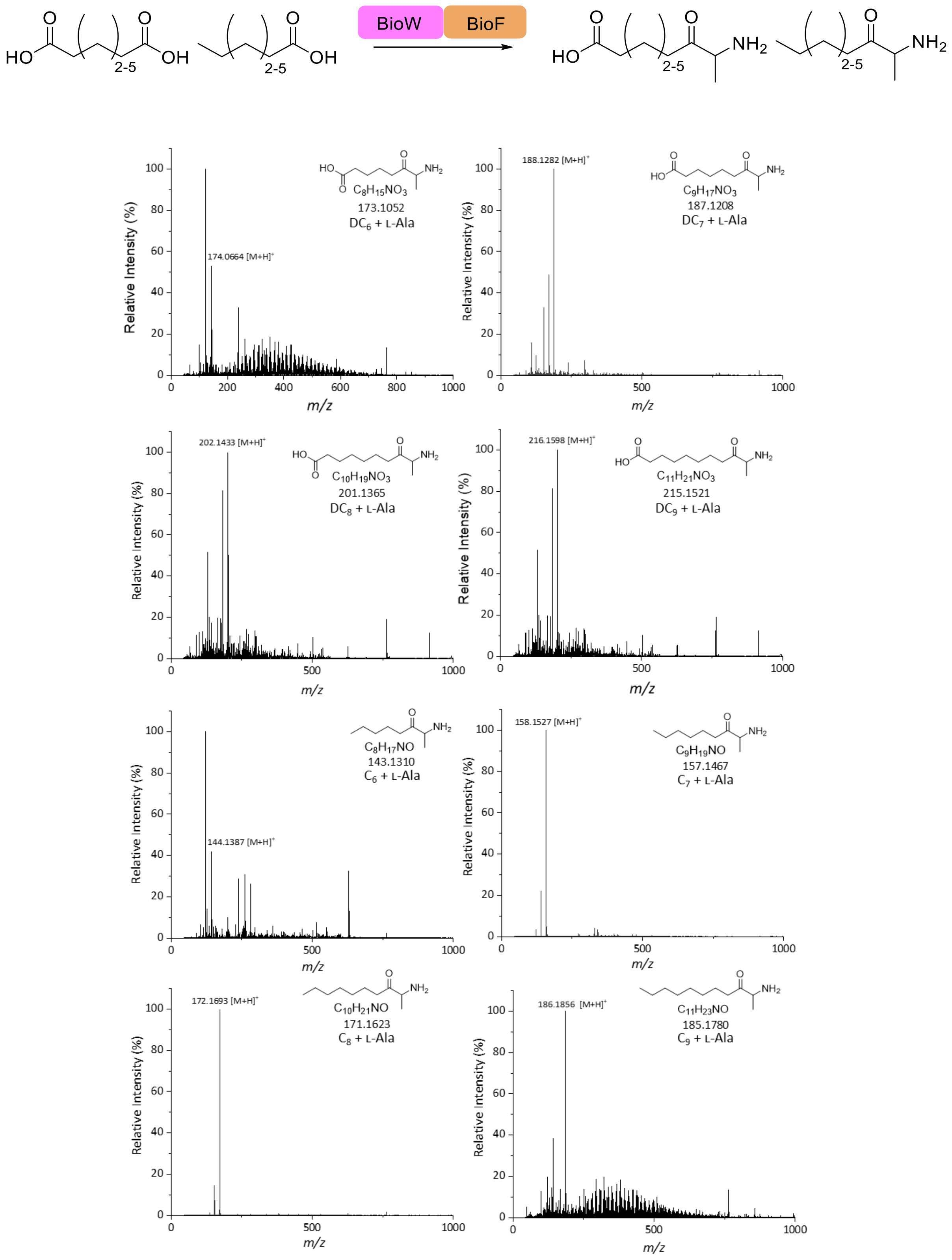

Figure. S20: Full *Ca*BioWF reactions with various di and mono-carboxylic acid chain lengths.
*Ca*BioWF reactions transforming a range of carboxylic acids (DC_6-DC_9 and C_6-C_9) with L-Ala leading to the production of the corresponding aminoketone. Product formation confirmed by LC ESI-MS analysis.

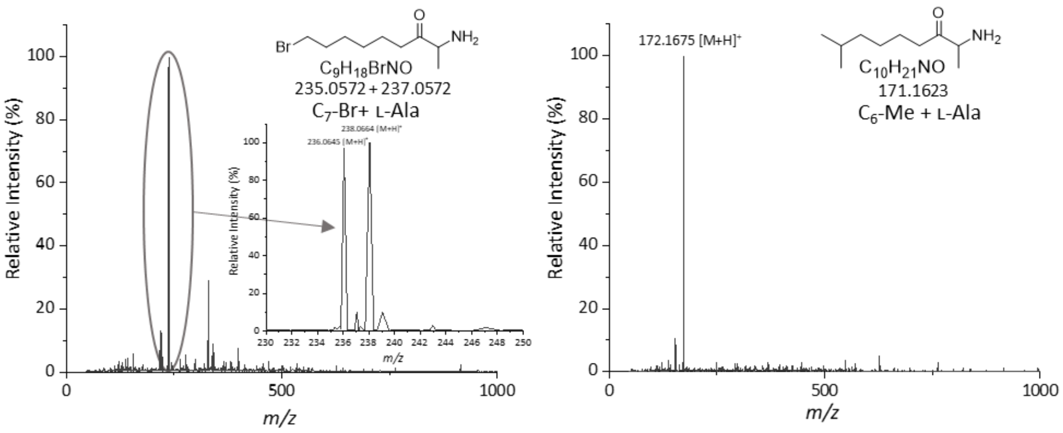

Figure. S21: *Ca*BioWF unusual carboxylic acid reactions

*Ca*BioWF catalysed formation of unusual AON analogues upon incubation of 7-Bromoheptanoic acid and 6-methylhexanoic acid with L-Ala. Product formation confirmed by LC ESI-MS analysis.

_Ca_BioWF Modelling and Simulation

Initially, the *Ca*BioW (M1-T238) and *Ca*BioF (G239-A620) domains were modelled separately using the accurate deep learning architecture ColabFold (see the Experimental Section in the main text). Both domains were predicted with high confidence (pLDDT >90, pTM >0.85, see figure S22A-B) with homodimeric interfaces comparable to experimentally solved structures including *Bs*BioW (PDB: 5FLL, see figure S23B) and *Ec*BioF (PDB: 1DJ9, see figure S24B). *Ca*BioW was modelled with a subdomain architecture shared by type IV ANL enzymes (see also PDB: 5TV5), wherein the catalytic C-terminal subdomain binds its substrates and the structural N-terminal subdomain comprises a dimer interface. The predicted *Ca*BioF shares strong fold-level similarity with several BioF homologues (PDB: 5JAY, 6ONN, 5VNX, 7S5M), as well as other PLP-dependent enzymes such as serine palmitoyltransferases (SPT, PDB: 3A2B, 2X8U) and 2-amino-3-ketobutyrate CoA ligases (KBL, PDB: 7V58, 3TQX, 7BXP). Furthermore, several highly conserved residues that define the binding pocket of each domain were identified by evolutionary conservation analysis, including Y181 and R194 in *Ca*BioW (figure S23C) as well as H380 and K483¬ in *Ca*BioF (figure S24C). This initial study provided confidence in the ability of ColabFold to accurately predict the tertiary and quaternary structures of the *Ca*BioWF domains.

The full *Ca*BioWF dimer was subsequently modelled, and the top-ranked output (pLDDT 91.8, pTM = 0.68, see figure S22C) was studied in a 10 ns (5 x 10^6 time steps) molecular dynamics simulation (MDS, see the Experimental Section in the main text). While the individual domains were confidently predicted on a fold-level, there was some uncertainty regarding the relative orientation of the two *Ca*BioW domains, in part due to the disordered intra-domain linker(s) tethering *Ca*BioW and *Ca*BioF together (figure S22C). The predicted *Ca*BioWF model suggests that both domains contribute towards the dimeric interface, and these interfacial contacts are maintained over the course of the MDS (see figure S25). In particular, the *Ca*BioWF complex is stabilised by an average of 36 ± 7 interfacial hydrogen bonds, the majority of which (52%) occur within 2.72-2.93 Å (figure S26A/B). While the average radius of gyration (Rg = 3.57 ± 0.02 nm, Rg max-min = 0.160 nm) suggests that the *Ca*BioWF complex is stable, pairwise RMSD analysis reveals that the bifunctional enzyme exhibits a moderate amount of conformational flexibility, with RMSDs as high as 6 Å occasionally observed (figure S26C-D). Root Mean Square Fluctuation (RMSF) and B-factor analysis identifies the intra-domain linker and the *Ca*BioW domains as the most mobile regions of the protein (figure S27 A-C). In fact, this linker is flexible enough to allow light orientational adjustment of the *Ca*BioW domains within the first 2 ns of the simulation, with one of the *Ca*BioW domains rotating approximately 18.5 ° inwards from the start of the trajectory (figure S27D). By the midpoint of the simulation, the *Ca*BioW domains had settled, and both *Bs*BioW and *Ec*BioF could be comfortably superimposed onto the *Ca*BioWF complex (figure S28). Interestingly, the *Ca*BioW and *Ca*BioF binding pockets face each other approximately 4.75 nm apart; the proximity and orientation of these binding pockets suggests that the pimeloyl-CoA product of *Ca*BioW can easily diffuse into active site of *Ca*BioF. Taken all together, this in silico study provides insight into the didomain architecture of *Ca*BioWF, and hints towards both its flexibility and the existence of a potential "tunnel" that can channel products from the *Ca*BioW domain to *Ca*BioF (figure S29). This makes *Ca*BioWF an attractive, curious and potentially challenging target for future crystallographic trials.

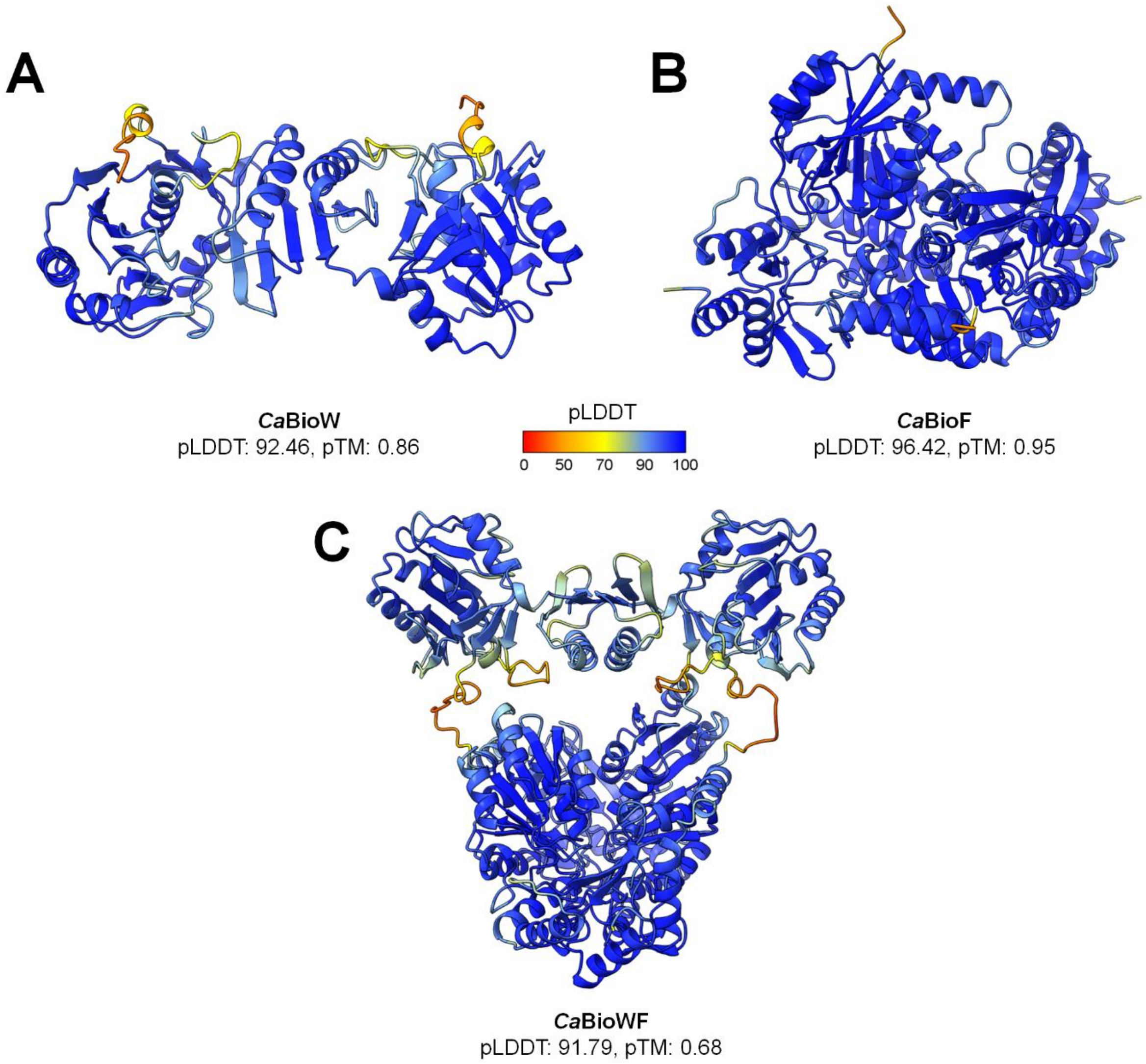

Figure. S22: pLDDT and pTM scores of the predicted CaBioWF domains

A) Confidence metrics for the predicted *Ca*BioW dimer. **B)** Confidence metrics for the predicted *Ca*BioF dimer. **C)** Confidence metrics for the predicted *Ca*BioWF dimer.

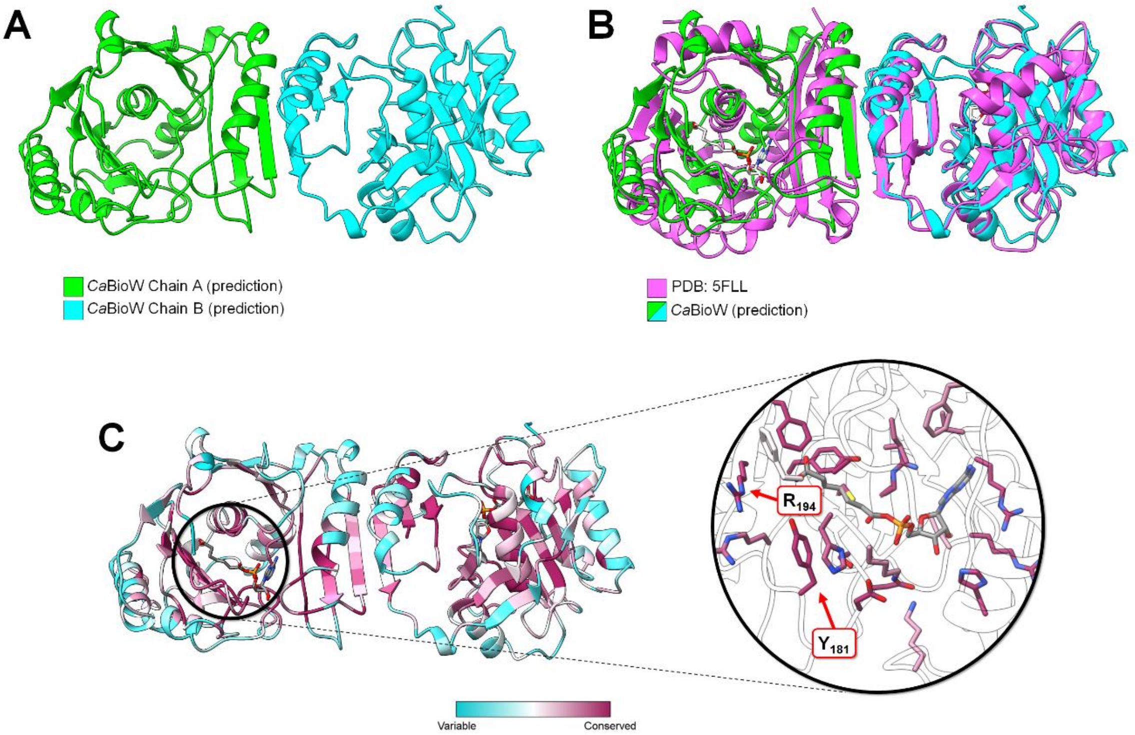

Figure. S23: A closer inspection of the predicted *Ca*BioW domain

A) *Ca*BioW was predicted to form dimeric contacts between the N-terminal subdomains. **B)** The crystal structure of *Bs*BioW superimposed on the predicted *Ca*BioW homodimer (RMSD: 1.06 Å, 174 pruned atom pairs). **C)** Highly conserved pocket residues identified in the predicted *Ca*BioWF. The pimeloyl adenylate was extracted from the *Bs*BioW crystal structure (grey, PDB: 5FLL) and is displayed here for reference.

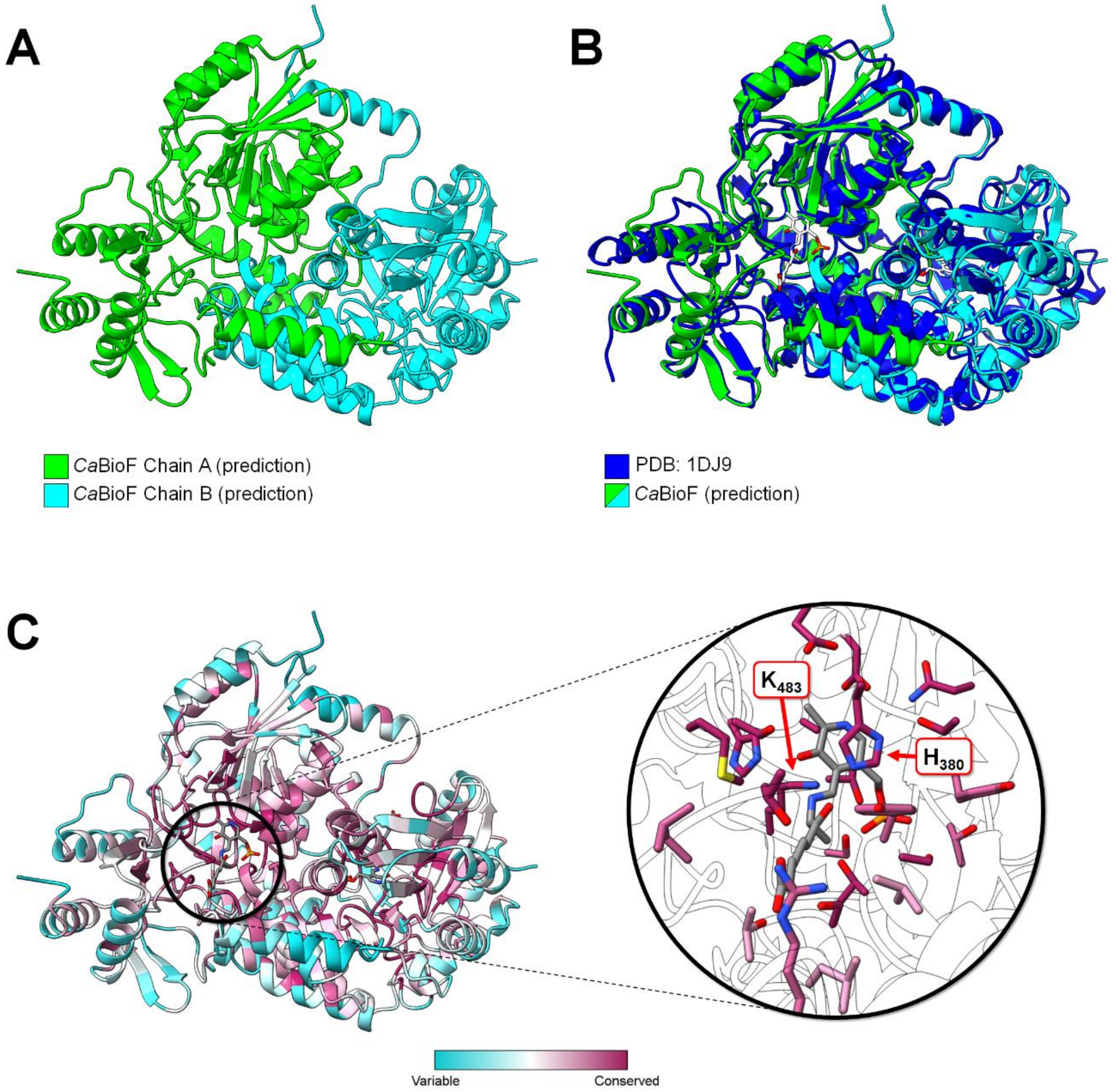

Figure. S24: A closer inspection of the predicted *Ca*BioF domain

A) *Ca*BioF was predicted to form dimeric contacts, as commonly observed with BioF homologues and other PLP-dependent enzymes. **B)** The crystal structure of *Ec*BioF superimposed on the predicted *Ca*BioF homodimer (RMSD: 0.99 Å, 289 pruned atom pairs). **C)** Highly conserved pocket residues identified in the predicted *Ca*BioF model. The AONS:PLP-AON product external aldamine was extracted from the *Ec*BioF crystal structure (grey, PDB: 1DJ9) and is displayed here for reference.

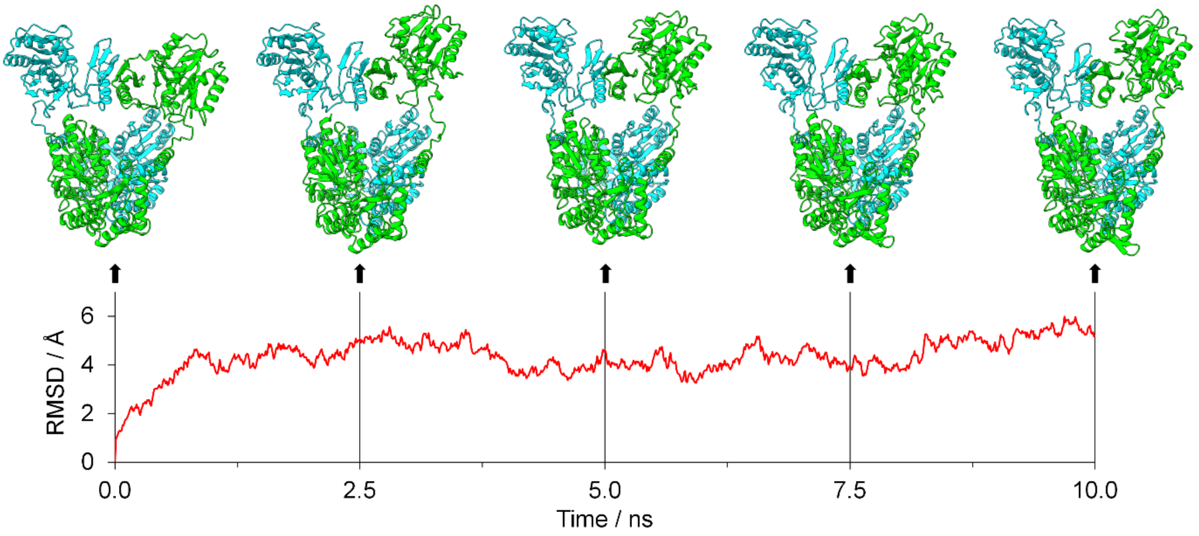

Figure. S25: Visualisation of the CaBioWF simulation

The displayed structures and RMSD plot (vs. t = 0 ns) depict the *Ca*BioWF morphology through time. Chains A and B are coloured lime and cyan respectively.

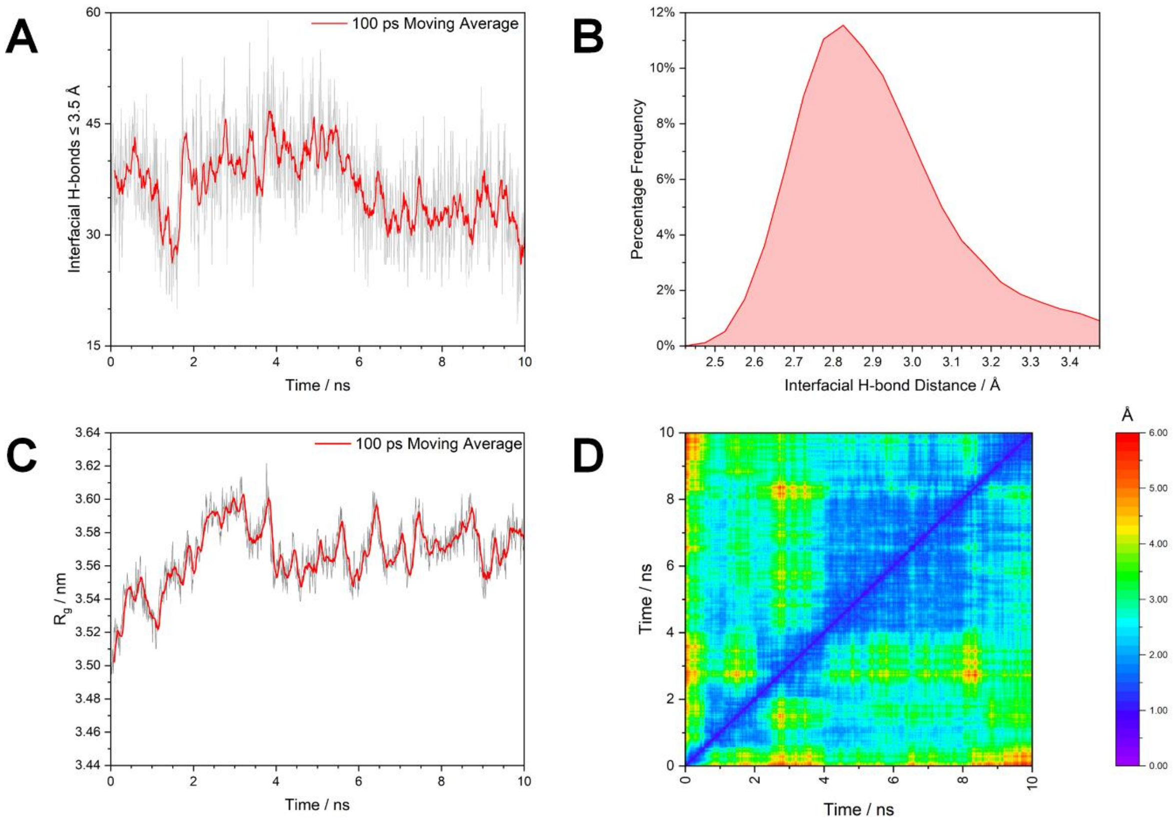

Figure. S26: A summary of the *Ca*BioWF MDS

A) An examination of the number of inter-chain hydrogen bonds over time. **B)** A distance distribution of the inter-chain hydrogen bonded contacts. **C)** The radius of gyration (Rg) of the *Ca*BioWF complex over time. **D)** A pairwise (2D) RMSD map computed between structures at every time point of the simulation.

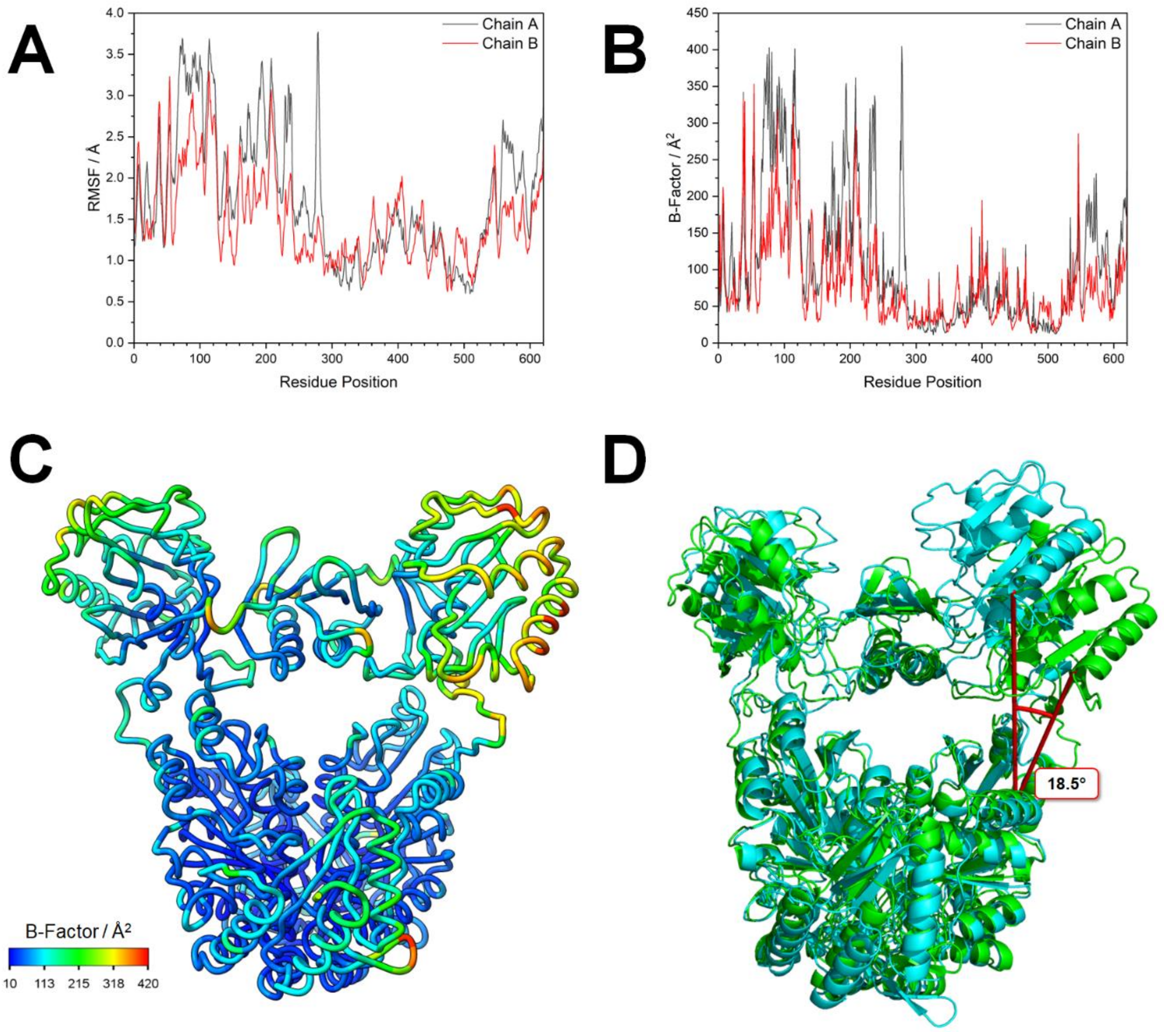

Figure. S27: Fluctuation analysis of the simulated CaBioWF complex

A) A per-residue root mean square fluctuation (RMSF) plot calculated for the Cα atoms of the protein. **B**) A per-residue B-factor plot. **C**) The per-residue B-factors computed in B mapped onto the *Ca*BioWF structure (pre-simulation). **D**) Superimposition of the t = 0 ns (lime) and t = 10 ns (cyan) structures, highlighting the mobility of the chain A *Ca*BioW domain; angles were calculated in PyMOL.

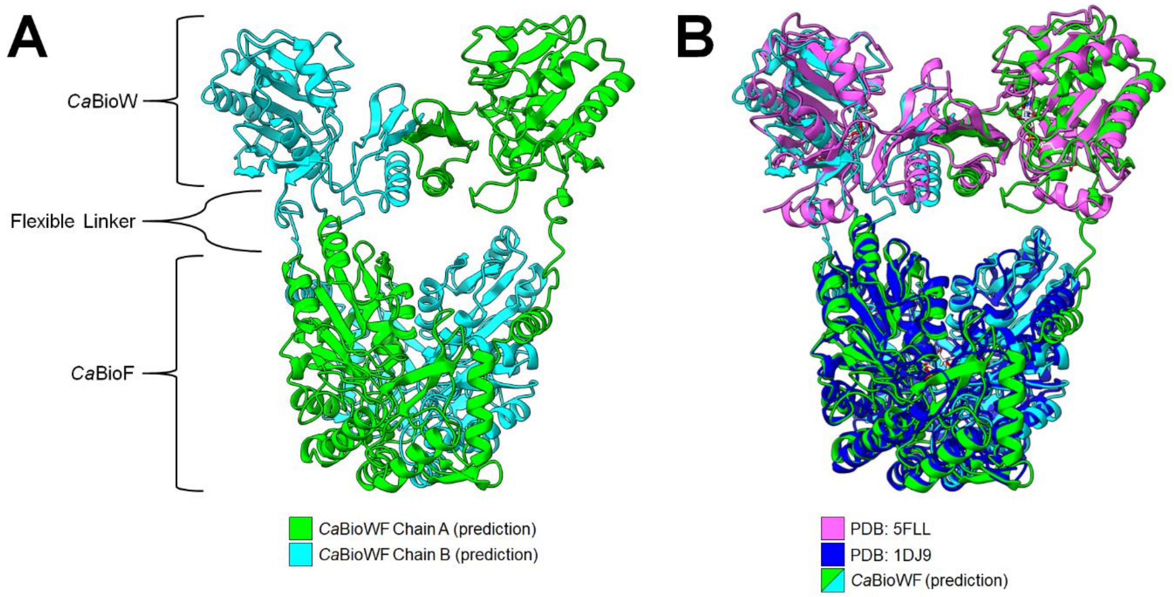

Figure. S28: The *Ca*BioWF complex sampled midway (t = 5 ns) through the MDS

At this timepoint, the *Ca*BioW domains have relaxed into a stable orientation. **A)** Annotation of the relaxed *Ca*BioWF complex. **B)** Superimposition of BsBioW (magenta, RMSD: 1.21 Å, 112 pruned atom pairs) and *Ec*BioF (blue, RMSD: 1.19 Å, 252 pruned atom pairs).

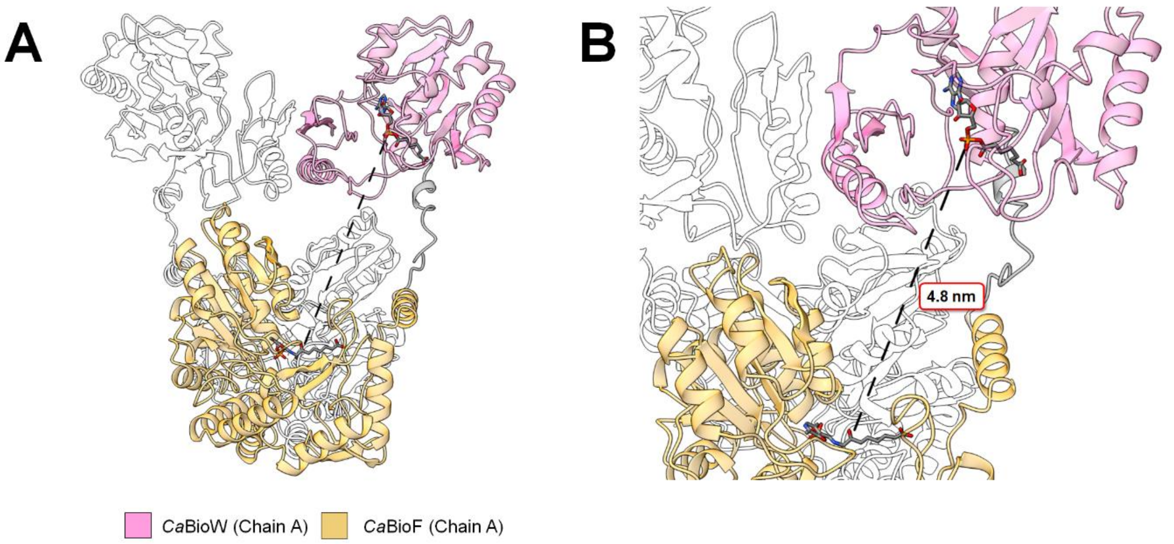

Figure. S29: A predicted molecular “tunnel” (dashed line) between the *Ca*BioW and *Ca*BioF domains. The structure was sampled at the midpoint (t = 5 ns) of the MDS. The ligands displayed (grey) were extracted from the *Bs*BioW and *Ec*BioF crystal structures and are displayed here for reference. **A)** The BioW and BioF binding pockets face towards each other, providing an easy diffusion path from *Ca*BioW domain to the active site of *Ca*BioF. **B)** The predicted “tunnel” is estimated to be 4.5-5 nm in length.
